# Modelling water and nutrient fluxes in the Danube River Basin with SWAT

**DOI:** 10.1016/j.scitotenv.2017.05.242

**Published:** 2017-12-15

**Authors:** Anna Malagó, Faycal Bouraoui, Olga Vigiak, Bruna Grizzetti, Marco Pastori

**Affiliations:** European Commission, Joint Research Centre (JRC), Directorate D – Sustainable Resources, Ispra, VA, Italy

**Keywords:** Swat, Danube, Streamflow, Nutrient concentrations, Nitrogen and phosphorus balances

## Abstract

This study provides an innovative process-based modelling approach using the SWAT model and shows its application to support the implementation of the European environmental policies in large river basins. The approach involves several pioneering modelling aspects: the inclusion of current management practices; an innovative calibration and validation methodology of streamflow and water quality; a sequential calibration starting from crop yields, followed by streamflow and nutrients; and the use of concentrations instead of loads in the calibration. The approach was applied in the Danube River Basin (800,000 km^2^), the second largest river basin in Europe, that is under great nutrients pressure. The model was successfully calibrated and validated at multiple gauged stations for the period 1995–2009. About 70% and 61% of monthly streamflow stations reached satisfactory performances in the calibration and validation datasets respectively. N-NO_3_ monthly concentrations were in good agreement with the observations, albeit SWAT could not represent accurately the spatial variability of the denitrification process. TN and TP concentrations were also well captured. Yet, local discrepancies were detected across the Basin. Baseflow and surface runoff were the main pathways of water pollution. The main sinks of TN and TP diffuse emissions were plant uptake which captured 58% of TN and 92% of TP sources, then soil retention (35% of TN and 2% of TP), riparian filter strips (2% both for TN and TP) and river retention (2% of TN and 4% of TP). Nitrates in the aquifer were estimated to be around 3% of TN sources. New reliable “state-of-the-art” knowledge of water and nutrients fluxes in the Danube Basin were thus provided to be used for assessing the impact of best management practices and for providing support to the implementation of the European Environmental Directives.

## Highlights

• A new SWAT model's calibration and validation method for water quantity and quality is provided.
• The approach includes prediction of concentrations as affected by current conservation measures.
• Modelled water and nutrient balances can guide conservation management.
• Prediction of nutrient concentrations increased the robustness of nutrient fluxes estimations.

## Graphical abstract

**Figure:**
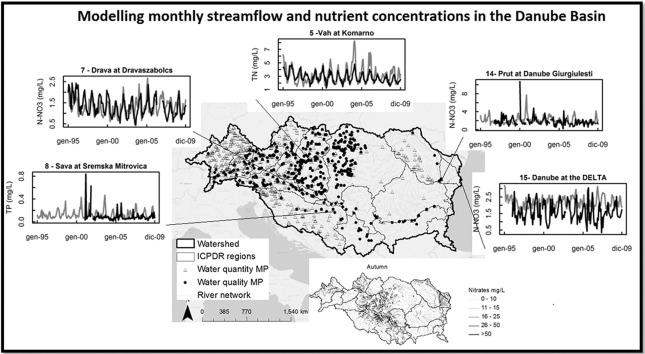

## 1 Introduction

In December 2000, the Water Framework Directive (WFD) of the European Union (EU) was enforced (EC, 2000) to provide a new legislative basis for water management, in terms of quantity and water chemical and ecological quality in Europe. According to the WFD, one of the purpose of EU Nitrates Directive (EC, 1991) and the Groundwater Directive (EC, 2006) is to keep nitrates concentration below a threshold of 50 mg/L. In this context, computer modelling systems provide an important contribution to the process of integrated management and decision support, in particular for establishing action plans and to assess the implementation of the measures and their impacts (Lindenschmidt et al., 2007, Abbaspour et al., 2015). Large-scale hydrologic and water quality models (H/WQ) are increasingly used for this purpose (Döll et al., 2008). In particular, Ferrant et al. (2011) indicated that several models have coupled hydrological and crop model to study the interaction between agricultural practices and catchment physical characteristics on the dynamic of nutrients in streams (i.e. Liu et al., 2005, Jha et al., 2006). Without being exhaustive, the H/WQ models commonly used at large-scale include SWAT (Arnold et al., 1998), WARMF (Herr and Chen, 2012), HSPF (Duda et al., 2012) and MIKE-SHE (Jaber and Shukla, 2012). Bouraoui and Grizzetti (2014) provide a more detailed review of models used in pollution assessment in Europe. The H/WQ have the capacity to represent appropriately spatial-temporal heterogeneity through a distributed or semi-distributed spatial discretization (Baffaut et al., 2015). The Soil and Water Assessment Tool (SWAT) (Arnold et al., 1998) has been extensively used for its modularity between quantity and quality components, computational efficiency, ability to predict long-term impacts as a continuous model (see for more details Gassman et al., 2010, Abbaspour, 2008, Di Luzio et al., 2005). A significant number of SWAT model applications has been reported ranging from subbasin to continental scales addressing different environmental issues (see overviews in Gassman et al., 2007, Gassman et al., 2014, Krysanova and Arnold, 2008, Douglas-Mankin et al., 2010, Tuppad et al., 2011, Krysanova and Srinivasan, 2015, Krysanova and White, 2015 and the most recent works of Haas et al., 2017 and Huang et al., 2017).

H/WQ models, such as SWAT, are particularly widely used in large transboundary river basins where many challenges exist due to different legislative frameworks and data availability across national boundaries (Chapman et al., 2016, Bloesch et al., 2012, Sommerwerk et al., 2010).

The Danube River Basin with a total area of about 800,000 km^2^ shared by 80 million people in 19 countries is recognized as the world's most international river basin. Management of water quantity and quality in the Danube Basin has been a top priority since decades (Liska, 2015). The Basin water management is coordinated by the International Commission for the Protection of the Danube (ICPDR) that has recently developed an updated Danube River Basin Management Plan (ICPDR, 2015) following the EU Water Framework Directive (EC, 2000) cycling approach. According to the Plan assessment, 49% and 35% of total 25,582 km of rivers in the Basin are at risk of failure to achieve the good ecological and chemical status by 2021 (ICPDR, 2015), mainly due to the high organic and nutrient pollution from point sources (i.e. wastewater treatment plant discharges) and diffuse sources (i.e. atmospheric deposition, excessive fertilization, and tile drainage systems), hazardous substances, and hydromorphological alterations. Furthermore, the rivers in the Basin are impacted by excessive water abstractions mainly for hydropower generation and irrigation use that can significantly alter the streamflow and consequently reduce available water resources.

To provide solutions to these issues, several H/WQ models were developed and applied in the Danube River Basin. Fehér and Muerth (2015) provide an exhaustive inventory of hydrological models applied in the Danube Basin giving details about models structure, spatial and temporal scales, and aims of the applications. Pagliero et al. (2014) applied the Soil and Water Assessment Tool model (SWAT; Arnold et al., 1998) to the Danube Basin delivering an accurate water resources assessment. Based on this study, Karabulut et al. (2016) developed a framework for mapping indicators of water scarcity, water availability and water use in the Danube Basin in the context of the ecosystem-water-food-energy nexus. Nutrients assessments have been conducted with the Modelling Nutrient Emissions into River Systems model (MONERIS; Behrendt and Opitz, 2000, Venohr et al., 2011) and the recent annual results of MONERIS for the period 2009–2012 were included in the 2015 Danube River Basin Management Plan. Both water quantity and quality aspects of the Black Sea Basin (2,300,000 km^2^) including the Danube River Basin were simulated using SWAT model by Rouholahnejad et al. (2014) and by Abbaspour et al. (2015). However, they focused mainly on calibration aspects at large scale rather than on water and nutrient balances of the Basin, which instead may offer important guidelines for management.

All these model applications provided valuable information on water and nutrient fluxes in the Basin but could be somehow limited in the representation of hydrological and water quality processes, since they failed to systematically address some drawbacks in water and nutrient modelling. A major risk is the exclusive focus on reaching very good fit between modelling results and observed water quantity and quality data with the risk to obtain a good fit for “wrong” reasons (Moriasi et al., 2015). For instance, good statistics can be obtained at gauging stations even though point sources are underestimated and the loads from agricultural lands are overestimated. This could result in policy scenarios that overestimate the impact of conservation or best management practices (BMPs) (Arnold et al., 2015). In addition, often the models were set-up without including the current management practices, irrigation, reservoirs, rivers and groundwater uses for which information are rarely available (Döll et al., 2009) and thus usually neglected. Furthermore, many water quality and quantity models are calibrated only at the final outlet of the watershed instead of using multiple gauging stations (Arnold et al., 2012b, Pohlert et al., 2007, Kuczera and Franks, 2002), or vice versa multiple gauging stations are involved in the calibration albeit affected by anthropogenic activities that the models are not able to represent (Pagliero et al., 2014). Finally, it has been observed that the above mentioned studies used the calculated nutrients load to calibrate the models albeit realistic estimation of loads is critical for accurate assessment of current water quality status (Lloyd et al., 2016). Measurement and analytical errors of concentrations are generally considered to be the smaller components of the total uncertainty associated with load estimation (Rode and Suhr, 2007). This high uncertainty has important implications in the ability to assess the effectiveness of management strategies which have to be implemented to mitigate diffuse agricultural water pollution.

For these limitations, further efforts in modelling water quantity and quality are necessary. Recently, the use of both soft and hard data (Arnold et al., 2015) was found to be most useful in analysing topics with missing data. Hard data are defined as long-term, measured time series, typically at a gauging station within a watershed, whereas soft data are defined as information on individual processes within a balance that may not been directly measured (i.e. ancillary data simulation from other models, GIS-map inspection, regional statistics and literature information). For instance, Yen et al. (2014) used soft data of denitrification rate from subsurface tile flow to constrain SWAT parameters related to denitrification process in a little Eagle Creek watershed (248 km^2^, United States). Vigiak et al. (2015) instead used soil loss rates measured on runoff plots from literature to calibrate the gross erosion in the Upper Danube (132,000 km^2^). To improve the robustness of calibration process, Pagliero et al. (2014) developed a step-wise calibration approach for streamflow based on a limited group of independent gauged subbasins (headwaters) considered more appropriate to represent the natural condition of subbasins. Selected parameters underpinning each hydrological process were systematically calibrated and then transferred to ungauged subbasins trough a regionalization technique. This approach was successfully applied in large regions, i.e. Danube (Pagliero et al., 2014), Scandinavia, Iberian Peninsula, Upper Danube and Crete Island (Malagó et al., 2015, Malagó et al., 2016, Malagó et al., 2015) decreasing the computation time for calibration at the large scale and gaining good knowledge and insights of each hydrological process.

The objective of this study was to develop a robust strategy of model calibration and validation, inspired by the efforts done in recent aforementioned studies, that addresses gaps observed in the international literature, and to apply the approach to the Danube River Basin, analysing in depth the hydrology and nutrients balance of this large transboundary basin. More specifically, this work aimed at (i) developing a robust modelling of water quantity and quality using both hard and soft data for the entire Danube Basin (800,000 km^2^) using SWAT; (ii) assessing current water and nutrient fluxes accounting for conservation measures including agricultural and water practices that are present across the Basin and that were not considered in previous studies (i.e. in Pagliero et al., 2014); (iii) providing long term mean annual water and nutrients balances for effective water management; and (iv) identifying hot spots of nitrate contamination that breach European drinking-water standards.

We start with briefly describing the Danube River Basin, and then introducing the structure, algorithms and set up of the SWAT model for the whole Danube. Next, we focus on water quantity and quality modelling approach, and in particular on the innovative aspects of the calibration and validation methodology. Then, we present water and nutrients fluxes across the Basin identifying regions and processes for which the model provides robust assessment, or conversely, where further investigations are necessary. Finally, hot spots of nitrate concentration in rivers are identified in the Basins and discussed.

## 2 Material and methods

### 2.1 The study area and data collection

The Danube River Basin is the second largest river basin in Europe, covering approximately 800,000 km^2^ of Central and South-Eastern Europe. In 2015, 19 countries were sharing the catchment, 14 of which are called ‘Danube countries’ (ICPDR, 2009).

Due to its vast area and its topography ranging from lowlands to mountains above 3000 m a.s.l., the Danube River Basin exhibits a pronounced climatic variability. The western region is influenced by the Atlantic climate, whereas the eastern region is characterized by a continental climate leading to lower precipitation and typically colder winters. The mean annual precipitation for the whole Danube basin was 597 mm/y for the period 1980 to 2009, ranging from 220 mm/y near the outlet of the river to 1510 mm/y in the Alps. The mean annual temperature for the period was 9.7 °C, ranging from 0.8 to 13 °C.

Dominant land cover types in the Basin are agriculture (42%) and forest (35%) (Karabulut et al., 2016). The rest of the basin is either covered by grasslands and heathlands (16%), urban areas (5%) and water bodies (< 2%) (EEA, 2013). The irrigated area is around 9000 km^2^ (only ~ 1% of arable land), and the volume of irrigation is approximately 3000·10^6^ m^3^ (Portmann et al., 2008).

**Fig. 1:**
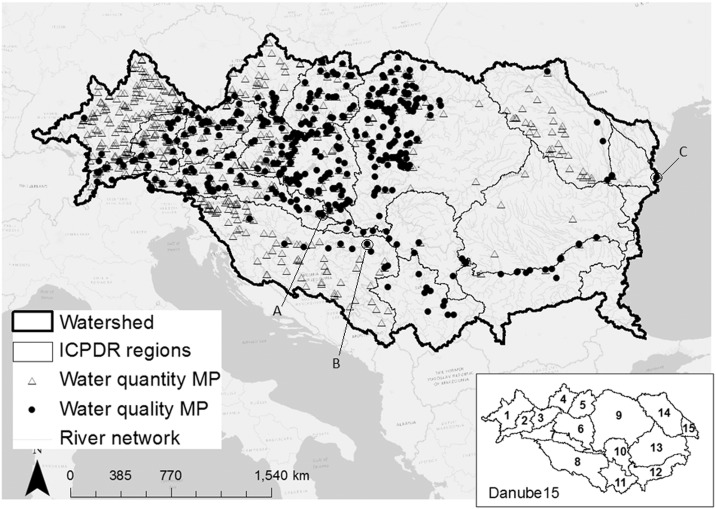
Location of the Danube River Basin in Europe (background map) with the monitoring points (MP) involved in this study, and the 15 ICPDR water management regions (insert): 1 = Upper Danube; 2 = Inn; 3 = Austrian Danube; 4 = Morava; 5 = Vah-Hron-Ipel; 6 = Pannonian Danube; 7 = Drava; 8 = Sava; 9 = Tisa;10 = Middle Danube; 11 = Velika Morava; 12 = Bulgarian Danube; 13 = Romanian Danube; 14 = Siret-Prut; 15 = Delta-Liman. The letters A–B–C indicate the location of three stations for which time-series of simulated discharge and nutrients in the rivers are provided in Fig. 4.

The Danube River can be divided into four general sections, the Upper, Middle, Lower Danube, and Delta (Habersack et al., 2013). Within these sections 15 water management regions were identified (DPRP Danube Pollution Reduction Programme, 1999, Vogel and Pall, 2002, ICPDR, 2009) (Fig. 1). Table 1 provides specific information for each region.

**Table 1:** Overview of the main characteristics of the 15 ICPDR water management regions of Danube Basin (ID: identification number of each region). The overview includes the model discretization in terms of Hydrological Response Units (HRUs), the geographic characteristics and the current management practices included in SWAT set-up of the Basin. The reported percentages for each region were calculated excluding upstream area.

ID	Name	SWAT implementation	Regional characteristics								Current management practices						
		HRUs	Area (km^2^)	Drain Area (km^2^)	Average elevation (m)	Average slope (%)	Long mean annual precipitation (mm)^a^	Cropland (%)	Forest (%)	Pasture (%)	Artificial Drainage (%)	Irrigated land (%)	Conservation tillage (%)	Cover crops (%)	Residue Management (%)	Terraces (%)	Riparian strips (%)
1	Upper Danube	343	50,617	50,617	566	8	950	44	37	18	8	0	11	4	2	29	0.4
2	Inn	157	25,999	25,999	1132	28	1200	8	51	41	2	0	3	1	0	43	1.1
3	Austrian Danube	142	25,187	101,803	621	18	1000	19	53	28	4	1	4	5	0	31	0.8
4	Morava	191	26,628	26,628	347	7	540	73	13	13	7	1	32	7	3	17	0.0
5	Vah-Hron-Ipel	210	30,587	30,587	385	11	720	39	40	21	19	3	8	1	3	32	0.0
6	Pannonian Danube	415	58,486	217,504	175	4	790	74	12	12	26	1	12	3	5	35	0.1
7	Drava	242	39,679	39,679	600	16	860	21	47	32	4	0	1	2	1	35	0.6
8	Sava	601	100,102	100,102	460	14	915	32	48	20	5	0	4	1	1	35	0.5
9	Tisa	958	149,567	149,567	303	9	590	50	33	16	18	1	7	2	3	29	0.1
10	Middle Danube	215	37,255	544,106	247	8	600	47	27	27	16	0	12	1	3	30	0.4
11	Velika Morava	214	37,702	37,702	574	16	790	48	30	22	1	0	4	0	3	37	0.3
12	Bulgarian Danube	272	46,307	721,734	307	9	570	65	16	19	3	1	36	0	1	23	0.7
13	Romanian Danube	562	93,619		272	7	570	59	17	23	1	5	4	1	2	20	0.1
14	Siret-Prut	412	68,012	68,012	339	10	560	40	27	33	0	1	5	1	2	25	0.4
15	Delta-Liman	96	13,917	803,663	49	3	710	72	0	27	9	0	5	3	2	100	0.1
	Danube15	5030	803,663	803,663	425	11	530	47	31	22	9	1	9	2	2	35	0.3

a Source: ESAF-meteo database (Ntegeka et al., 2013) considering the whole drained area of each region.

### 2.2 The SWAT model

The Soil and Water Assessment Tool (SWAT; Arnold et al., 1998) was used to simulate water quantity and quality fluxes in the Danube Basin. SWAT is a process based, semi-distributed, basin-scale model that has been widely used in many large basins around the world (Gassman et al., 2014). There were two main reasons for selecting the SWAT model for this research: it has a flexible structure that allows addressing different water resource and pollution problems; and it is well documented and is an open source code that can be adapted to specific applications (Gassman et al., 2007).

The model operates at daily time step and its major components include weather, soil, hydrology, plant growth, nutrient cycles, and land management (Gassman et al., 2007, Arnold et al., 2012a). In SWAT, a watershed is divided into subbasins, which are further subdivided into hydrologic response units (HRUs) that consist of unique combinations of soil, land use/cover, and slope. The SWAT model structure comprises two phases: a land phase solved at HRU level, and a stream phase solved at reach level (Neitsch et al., 2011). In the land phase, the HRU water, sediment and nutrients cycles into soil, and losses are aggregated at the subbasin level. The movement of water, sediments, and nutrients through the stream network to the watershed outlet are instead simulated in the routing (stream) phase.

Table 2 summarizes the main water and nutrient processes and the corresponding SWAT algorithms used in this study. Sediment processes are presented in a companion paper (Vigiak et al., 2017).

**Table 2:** Main simulated processes and related algorithms implemented using SWAT in this study.

Component	Process	Algorithms	Reference
Hydrology	Surface runoff	SCS Curve Number method	USDA Soil Conservation Service (1972)
Hydrology	Lateral flow	Kinetic storage method	Neitsch et al. (2011); Malagó et al. (in press)
Hydrology	Baseflow	Baseflow recession constant; groundwater storage; re-evaporation	Neitsch et al. (2011)
Hydrology	Potential Evapotranspiration	Penman-Monteith	Monteith, 1965, Penman, 1956
Hydrology	Channel routing	Variable storage routing method	Williams (1969); Neitsch et al. (2011)
Hydrology	Tile drainage flow	Houghoudt and Kirkham drainage equations and drainage volume converted in water table depth using a variable water table factor	Moriasi et al., 2007a, Moriasi et al., 2013a, Moriasi et al., 2013b
Plant Growth	Biomass and crop yields	EPIC model equations	Williams et al. (1984)
Quality	Nitrogen and phosphorus cycle	Loading function; equations from Epic model, PAPRAN mineralization model	Neitsch et al. (2011); McElroy et al. (1976); Williams and Hann (1978); Van Keulen (1981)
Quality	Nitrate movement and soluble phosphorus movement	Exponential decay weighting function for transport in aquifer	Neitsch et al. (2011); Venetis (1969); Sangrey et al. (1984)
Quality	In stream nutrient-processes	QUAL2E model	Brown and Barnwell (1987)
Quality	Nutrients in water bodies	Nitrogen and phosphorus mass balance	Chapra (1997)
Quality	Riparian filters	Empirical equations based on runoff reduction ()	White and Arnold (2009); Neitsch et al. (2011)
Quality	Nitrogen and phosphorus in tile drainage system	Equations function of tile flow (m^3^/d), concentration in solution in the layer containing the tile drain and the percolation coefficient	Neitsch et al. (2011); Moriasi et al. (2013b)
Quality	Nitrogen and phosphorus from urban areas	build up/wash off approach	Huber and Dickinson (1988)

### 2.3 Model set up

The SWAT model mainly requires input data related to topography, land use, soil, climate, and land management. The Danube Basin was divided into 4663 subbasins based on the 100 m pixel size Digital Elevation Model CCM2 DEM (Vogt et al., 2007). The land use was defined using a map of 1 × 1 km for year 2000, built from the combination of CAPRI (Britz, 2004), SAGE (Monfreda et al., 2008), HYDE 3 (Klein Goldewijk and van Drecht, 2006) and GLC (Bartholome and Belward, 2005) databases. Crops were attributed to arable land subbasins according to European statistics at NUTS2 level (administrative subdivision) (EUROSTAT, 2013). Soil information was based on the 1 × 1 km Harmonized World Soil Database (HWSD) (FAO, 2008), using top soil layer data. Based on the combination of landuse, soils and slope, the Danube Basin was discretized into 5181 HRUs with a median area of 129 km^2^.

The nutrient emissions from point sources were retrieved from the UWWTD database (ICM, 2011) aggregated at subbasin level. This database reports the collected annual discharged nutrients loads from urban waste water treatment plants for the period 2007–2008 for the EU Member State. The aggregated values at subbasin level were kept constant for the whole simulation.

The climate data, including daily precipitation, temperature, solar radiation, wind speed and relative humidity, were obtained from EFAS-METEO at spatial resolution of 5 km × 5 km (Ntegeka et al., 2013) for the period 1990–2009. To account for the increase in precipitation with elevation that is typically observed in mountainous regions, four elevation bands were implemented.

Reservoirs and lakes exceeding 20 km^2^ (Lehner and Döll, 2004, Vogt et al., 2007) and hydropower plants of large generation capacity (> 10 MW; ICPDR, 2013) installed on the main rivers were also included in the model. The total extended area of these water bodies was around 2170 km^2^, < 1% of entire Basin, and their volumes were set according to Lehner and Döll (2004) and Vogt et al. (2007).

In this study land management data included agricultural practices that were not considered in previous studies. The main crop management operations consist of planting, fertilization, irrigation, tillage and harvesting. The timing of management operations was implemented through daily heat unit method (Arnold et al., 1998). In this study the heat units for each crop were calculated by Bouraoui and Aloe (2007). The application rates of manure and mineral fertilization was retrieved from the CAPRI model (Britz, 2004) for the year 2000 and kept constant through the simulation.

Irrigated cropland areas were identified using the MIRCA database (Portmann et al., 2008). About 9200 km^2^ of the Basin are irrigated (1% of entire Danube Basin), corresponding to a total of 290 HRUs. The SWAT auto-irrigation option, whereby irrigation is applied when the soil moisture content drops below a threshold value, was used. Conventional tillage was implemented on HRUs with annual crops setting the plowing depth at 25 cm with biological mixing efficiency of 0.85 and harrowing at 7 cm, with mixing efficiency of 0.3.

Current extent of best management practices such as conservation tillage, cover crops and residue management in the Basin was based on Eurostat (2010) data. Based on this information, conservation tillage is applied in about 77,470 km^2^ in the Basin corresponding to 289 HRUs of annual crops. Conservation tillage consisted of harrowing at 7 cm with biological mixing efficiency of 0.4 since biological mixing can be significant in systems where the soil is less disturbed (Arnold et al., 2012b, Lam et al., 2011).

Cover crops extended over an estimated area of 15,013 km^2^, while residue management is used in 18,400 km^2^. Implementation in SWAT was set according to Arabi et al. (2007). The fraction of terraced pasture and permanent crop land was based on presence of stone walls per farm holding (Eurostat, 2010) using an approach similar to that applied by Panagos et al. (2015). The extent of terraces was estimated at about 2565 km^2^ and they were modelled following SWAT recommendations (Neitsch et al., 2011).

Riparian land was estimated using pan-European maps (Clerici et al., 2013, Weissteiner et al., 2013). Riparian filters were applied to agricultural HRUs following the method of White and Arnold (2009), as described in Vigiak et al. (2016).

According to the global artificially drained agricultural areas map (Feick et al., 2005), about 65,000 km^2^ (8% of the Basin) were identified as artificially drained. As a result, tile drainage was applied in 470 HRUs of flat cropland with poorly or moderately well drained soils.

The daily nitrogen atmospheric deposition is computed by SWAT in each subbasin based on the daily precipitation amount and the average nitrogen concentration in precipitation. This average nitrogen concentration was set at 1.8 mg/L accordingly to the EMEP data for the period 1995–2005 (EMEP, 2001).

Finally, the initial concentration of nitrates in the shallow aquifer was based on measurements in bore holes (EU, 2013). Nutrient concentrations in reservoirs were based on concentrations measured in gauging stations located immediately downstream.

The simulation period was 1995–2009 (15 years), in addition to 5 years of warm-up to initialize model variables and allow processes to reach a dynamic equilibrium (Daggupati et al., 2015).

### 2.4 The calibration and validation of the model

In line with recent guidelines for water quantity and quality modelling (Arnold et al., 2015, Baffaut et al., 2015, Daggupati et al., 2015), a systematic calibration and validation (C/V) approach is proposed and applied in the Danube River Basin. It includes a robust and reproducible C/V strategy using both hard and soft data. The proposed C/V strategy is a sequential approach since each step is influenced by the previous one (Malagó et al., 2015). It involves four modules: (i) module 1, “crop yield”, that involves the calibration of annual crop yields; (ii) module 2, “hydrology”, that focuses on the calibration/validation of streamflow (and its components) and the extrapolation of streamflow to ungauged subbasins; (iii) the module 3, “sediment”, that consists of the calibration/validation of gross erosion and annual sediment concentrations; and finally (iv) the module 4, “nutrients”, that consists of the calibration of denitrification process in the soil and the calibration/validation of monthly concentrations of nitrate-nitrogen (N-NO_3_), total nitrogen (TN) and total phosphorus (TP).

Modules 1, 2 and 4 of the C/V are described in detail in this paper, while the sediment module is described in Vigiak et al. (2017).

The parameters selected for the calibration of each module were based on a literature search (i.e. van Grievensen et al., 2006, Santhi et al., 2001, Grizzetti et al., 2015, Me et al., 2015, Haas et al., 2015, Yen et al., 2014, Cerro et al., 2012, Omani et al., 2012, Chu et al., 2004), the main processes involved and a preliminary global sensitivity analyses, performed using Latin Hypercube (LH) sampling methods using the SWAT-CUP program (Abbaspour, 2007).

#### 2.4.1 Monitoring data

An extensive database of streamflow and nutrient concentrations (mg/L) in the Danube River Basin was assembled from several sources (Table 3) covering the period 1995–2009 (15 years).

**Table 3:** Streamflow and nutrients data collected in the Danube Basin (# = number; Data type: Q = streamflow (m^3^/s), N-NO3 = nitrogen nitrates (mg/L); TN = total nitrogen (mg/L), TP = total phosphorus (mg/L); MP = monitoring points).

Acronym	Data provider and owner	Data type	Time step	#MP	#data entries	Period extent	
ATR	Austrian Environment Agency (http://www.umweltbundesamt.at)	Q	Daily	151	824,723	1995	2009
		N-NO3	Daily	106	10,913	1995	2009
		TP	Daily	106	10,817	1995	2009
BAFU	Swiss Federal Office for the Environment (http://www.bafu.admin.ch/hydrologie/index.html?lang=en)	Q	Daily	1	5479	1995	2009
CZR	Czech Hydrometeorological Institute (http://hydro.chmi.cz/ismnozstvi/)	Q	Monthly	16	983	2004	2009
EWA	http://www.ewa-online.eu/	Q	Daily	25	135,297	1995	2009
HUWQ	Hungarian General Directorate of Water Management (http://www.ovf.hu/en/) BME (http://www.vkkt.bme.hu/munkatars/?mid=10)	Q	Daily	118	587,525	1995	2009
		N-NO3	Daily	183	43,173	1995	2009
		TN	Daily	149	17,415	1995	2009
		TP	Daily	182	41,317	1995	2009
ICPDR	International Commission for the Protection of the Danube River (http://www.icpdr.org/wq-db/)	Q	Daily	5	11,689	1995	2009
		N-NO3	Daily	2	498	1996	2009
		TN	Daily	2	272	2000	2009
		TP	Daily	2	370	1996	2009
JRC	JRC (European Commission, Joint Research Centre) database	Q	Daily	112	531,012	1995	2009
		N-NO3	Daily	57	9870	1996	2009
		TN	Daily	43	3434	1995	2009
		TP	Daily	51	7916	1996	2009
LFU	Bavarian Environment Agency (http://www.lfu.bayern.de/index.htm)	Q	Daily	103	556,640	1995	2009
SAVA	International Sava River Basin Commission (http://www.savacommission.org/)	Q	Daily	45	94,791	1995	2009
RSEPA	Serbian Environmental Protection Agency (http://www.sepa.gov.rs/index.php)	N-NO3	Daily	13	1897	1996	2009
		TN	Daily	13	484	2002	2009
		TP	Daily	13	1136	1996	2009
SIRET	University of Suceava, Romania (Radoane et al., 2013)	Q	Daily	32	173,493	1995	2009
SK	Slovak University of Technology in Bratislava	Q	Daily	62	318,696	1995	2009
		N-NO3	Daily	55	6753	1995	2009
		TN	Daily	53	2711	1995	2009
		TP	Daily	55	6589	1995	2009
SLV	Slovenian Environment Agency (http://vode.arso.gov.si/)	Q	Daily	38	192,329	1995	2009

The streamflow dataset involved 708 monitoring points. It was the richest in terms of spatial and temporal extent, whereas the nutrients datasets consisted of 416 monitoring points for N-NO_3_, 260 of TN and 409 of TP, albeit the data collected reported both mineral and organic forms of nitrogen and phosphorus. The sampling frequency was usually once a month, and sometimes samples in large river cross sections were taken at three or more locations (i.e. on the left, in the middle or on the right bank of the river). In these cases, the average concentration of all samples was retained. In addition, an extensive data quality check of these measurements was performed to remove unrealistic values (due to typing errors for instance) and correct for heterogeneous units. We use the statistical software R (R Development Core Team, 2008) for calculating the main descriptive statistics (25th and 75th percentiles, median and mean) for each variable and for automatically generating plots of the time series of total nitrogen and nitrogen-nitrate (N-NO_3_), as well as total phosphorus and phosphates (P-PO_4_) for each station. Using both the descriptive statistics and the plots we identified the stations with inconsistent observed nutrients values (i.e. anomalies in the units of measurement and conversions). A manual correction of the anomalies was then applied for each of the selected stations. Finally, the descriptive statistics and plots were regenerated to check the consistency of the datasets.

Concentration at the Danube outlet was calculated as the average concentration monitored in the three main arms of the Delta. Given the heterogeneous spatial distribution of nutrient observations in the Basin, the whole dataset of nutrient concentrations was used for calibration, whereas monthly loads were employed in the final evaluation of the model. The loads were calculated using nutrients concentrations and daily streamflow based on the flow weighted concentrations method proposed by Moatar and Meybeck (2005).

#### 2.4.2 Crop yields calibration

SWAT simulated mean annual crop yields were compared with those reported by EUROSTAT (EUROSTAT, 2013) for each Danube Country. To perform the comparison, the predicted crop yield was converted to fresh weight yield by using a conversion table from the EPIC model (Williams et al., 1984). The calibration was performed manually changing the crop harvest index (HVSTI), the optimal and minimum temperature plant growth (T_OPT, T_BASE) (Table 4). The visual appraisal of calibrated and simulated annual crop yields was used as criterion to define the near optimal parameter values.

**Table 4:** Parameters involved in the calibration with their range before and after calibration. The values in the bracket represent the average of calibrated values. For each parameter the related SWAT input file, the method of calibration (M = manual; SA = semi-automatic calibration using SUFI-2), and type of data used for calibration are reported.

Module	Process	Parameter and input file	Definition	Unit	Range	Calibrated Range (average value)	Data used in calibration	Calibration method
Crop yields	Plant growth	HVSTI.crop	Crop harvest index for optimal growing conditions	NA	0.02–2	0.04–2.5 (0.68)	EUROSTAT (2013)	M
	Plant growth	T_OPT.crop	Optimal temperature for plant growth	^o^C	12.5–30	12.5–30 (22.6)	EUROSTAT (2013)	M
	Plant growth	T_BASE.crop	Minimum/base temperature for plant growth	^o^C	0–12	0	EUROSTAT (2013)	M
Water	Baseflow	ALPHA_BF.gw	Baseflow alpha factor	d	0–1	0.26–0.98 (0.73)	Monthly streamflow	SA
	Surface runoff	CN2.mgt1	SCS runoff curve number for moisture condition II	NA	− 15–+15 e	− 15–+15 (10)	Daily surface runoff	SA
	Surface runoff	CH_N1.sub	Manning's value for tributary channel	NA	0.025 - 0.30	0.01–0.14 (0.096)	Daily surface runoff	SA
	Tile drainage flow	DDRAIN.mgt1^a^	Depth to subsurface tile	mm	0–2000	300–900 (687)	Monthly streamflow	SA
	Tile drainage flow	DEP_IMP.hru^a^	Depth to impervious layer	mm	0–6000	1050–6000 (3100)	Monthly streamflow	SA
	Lateral flow	EPCO.hru	Plant evaporation compensation factor	NA	0.01–1	0.01–0.94 (0.44)	Daily lateral flow	SA
	Lateral flow	ESCO.hru	Soil evaporation compensation factor	NA	0.01–1	0.03–0.99 (0.57)	Daily lateral flow	SA
	Tile drainage flow	GDRAIN.mgt1^a^	Drainage lag time	hr	0–100	1–40 (20)	Monthly streamflow	SA
	Baseflow	GW_DELAY.gw	Groundwater delay	d	0–500	0.75–498 (41)	Monthly streamflow	SA
	Baseflow	GWQMN.gw	Threshold depth of water in the shallow aquifer required for return flow to occur	mm	0–1000	5.5–991 (618)	Monthly streamflow	SA
	Baseflow	GW_REVAP.gw	Groundwater ‘revap’ coefficient	NA	0.02–2	0.02–0.19 (0.06)	Monthly streamflow	SA
	Snow proecess	PLAPS.sub	Precipitation laps rate	mm/km	0–100	0.97–64.7 (24)	Monthly streamflow	SA
	Baseflow	RCHRG_DP.gw	Groundwater recharge to deep aquifer	fr	0–1	0.005–0.93 (0.09)	Monthly streamflow	SA
	Baseflow	REVAPMN.gw	Threshold depth of water in the shallow aquifer for revap to occur	mm	0–500	0.25–443 (196)	Monthly streamflow	SA
	Snow melt	SFTMP.sno	Snowfall temperature	^o^C	− 5–+5	− 1.57–1.11 (− 0.84)	Monthly streamflow	SA
	Snow melt	SMFMN.sno	Melt rate for snow on Dec 21	mm H_2_O °C^−^ 1 d^− 1^	0–10	0.09–9.66 (5.10)	Daily streamflow	SA
	Snow melt	SMFMX.sno	Melt rate for snow on Jun 21	mm H_2_O °C^−^ 1 d^− 1^	0–10	0.01–9.97 (4.36)	Daily streamflow	SA
	Snow melt	SMTMP.sno	Snowmelt base temperature	^o^C	− 5–+5	− 0.17–2.53 (0.60)	Monthly streamflow	SA
	Lateral flow/Infiltration	SOL_AWC.sol	Available water capacity of the soil layer	fr	− 25–+25	− 25–+25 (10)	Daily lateral flow	SA
	Lateral flow/Infiltration	SOL_K.sol	Saturated hydraulic conductivity	mm h^− 1^	− 25–+25	− 25–+25 (16)	Daily lateral flow	SA
	Snow melt	TIMP.sno	Snow pack temperature lag factor		0.01–1	0.01–0.55 (0.18)	Monthly streamflow	SA
	Snow melt	TLAPS.sub	Temperature laps rate	°C/km	− 10–0	− 9.82 to − 1.83 (− 5.37)	Monthly streamflow	SA
	Tile drainage flow	RE.hru/.sdr	Effective radius of drains	mm	5^d^–100	5–100 (52)	Monthly streamflow	SA
	Tile drainage flow	SDRAIN.hru/.sdr	Distance between two drain or tile tubes	mm	7600–30,000	5060–27,700 (16020)	Monthly streamflow	SA
	Tile drainage flow	DDRAIN_CO.hru/.sdr	Drainage coefficient	mm/day	10–51	6–50 (24)	Monthly streamflow	SA
	Tile drainage flow	LATKSATF.hru/.sdr	Multiplication factor to determine lateral ksat from SWAT ksat input value	NA	0.01–4	0.09–3.4 (1.22)	Monthly streamflow	SA
Nutrients	Denitrification	CDN.bsn	Denitrification exponential rate coefficient	NA	0–3	2.5	Velthof et al. (2009)	M/SA
	Mineralization	CMN.bsn	Rate factor for humus mineralization of active organic nitrogen	NA	0.0001–0.0003	0.000145	Monthly concentration	M/SA
	Transport of nitrogen with sediment	ERORGN.hru	Organic nitrogen enrichment ratio	NA	0–5	0.05–4.5 (0.7)	Monthly concentration	M/SA
	Shallow aquifer nitrates	HLIFE_NGW.gw	Half-life of nitrate–nitrogen in the shallow aquifer	day^− 1^	0–200	0–200 (116)	Monthly concentration	M/SA
	Nitrogen percolation	NPERCO.bsn	Nitrogen percolation coefficient	NA	0–1	0.5	Monthly concentration	M/SA
	Nitrogen settling rate	NSETLR1.lwq = NSETLR2.lwq^b^	Nitrogen settling rate	m/year	1 - 150^c^	5.5–150 (30)	Monthly concentration	M/SA
	Nitrogen uptake	N_UPDIS.bsn	Nitrogen uptake distribution parameter	NA	1–31	28	Monthly concentration	M/SA
	Residue	RSDCO.bsn	Residue decomposition coefficient	NA	0.02–1	0.02	Monthly concentration	M/SA
	Denitrification	SDNCO.bsn	Denitrification threshold water content	NA	0–1	1	Velthof et al. (2009)	M/SA
	Transport of phosphorus with sediment	ERORGP.hru	Organic phosphorus enrichment ratio	NA	0–5	0–0.25 (0.1)	Monthly concentration	M/SA
	Phosphorus settling rate	PSETLR1.lwq = PSETLR2.lwq^b^	Phosphorus settling rate	m/year	1–150^c^	9.5–150 (57)	Monthly concentration	M/SA

a Only in tile drained HRUs

b The nitrogen and phosphorus settling rate didn't change during the year.

c The range of settling rate of nutrients in reservoirs was set larger that the default accordingly with Panuska and Robertson (1999).

d The lower limit of the RE was set to 5 mm to investigate all the possible range of values

e CN2 was set to 30 in the HRUs with tile drain systems

#### 2.4.3 Streamflow calibration and validation

The calibration of streamflow and its components was performed using a step-wise approach that consists of a multi-variables calibration of headwater subbasins and a regionalization of the calibrated parameters (Pagliero et al., 2014, Malagó et al., 2015, Malagó et al., 2015). The calibration of streamflow and its components focuses on a limited group of independent gauged headwaters subbasins, since they are more likely to represent natural hydrological behaviour (Gudmundsson et al., 2012) and their streamflow components are more representative than larger basins where streamflow is often influenced by human activities (Döll et al., 2008).

The dataset of daily streamflow gauging stations was thus divided into a calibration dataset and a validation dataset with different spatial and temporal distribution: 264 stations with daily values for the period 1995–2006 were used for model calibration, while the validation dataset comprised the remaining 444 stations for 1995–2009 plus the 264 calibration stations for the period 2007–2009.

The daily streamflow of the 264 headwater calibration subbasins was divided into its main components (surface runoff SR, lateral flow LF, and baseflow BF) using the SWAT filter (Lyne and Hollink, 1979). The filter was applied twice, first it was applied to daily streamflow to separate baseflow from quick flow, and then to the quick flow to separate lateral flow from surface runoff. The streamflow components were calibrated separately using the software SWAT-CUP and SUFI-2 method (Abbaspour, 2007) in four sequential steps that focused on different hydrological processes: snow processes, surface runoff, lateral flow, and baseflow. A fifth final step was also performed by calibrating all hydrological parameters together using reduced ranges to account for any covariance of parameters belonging to different hydrological groups (Malagó et al., in press).

The final sets of calibrated parameters of subbasins that reached “acceptable performance” (Moriasi et al., 2007b) were transferred to ungauged subbasins using a regionalization technique coupled with a classification procedure based on hydrologic similarity. The method is described in detail in Malagó et al. (in press).

#### 2.4.4 Nutrient calibration and evaluation

The calibration of sediments (Vigiak et al., 2017) was followed by the calibration of nutrients which was based on concentrations rather than loads (usually performed in other studies) to avoid uncertainty issues related to loads estimation.

The mean soil denitrification (kg/ha/year) was calibrated for the whole Basin. In this study the annual denitrification was constrained using data obtained from the integrated assessment tool MITERRA-EUROPE (Velthof et al., 2009, Oenema et al., 2007). The parameters CDN (denitrification exponential coefficient) and SDNCO (denitrification threshold water content, or the fraction of field capacity water content above which denitrification is assumed to occur) were calibrated manually to fit the MITERRA-EUROPE values.

TN and TP concentrations were calibrated adjusting the selected parameters reported in Table 4. The parameters were selected based on a global sensitivity analysis that comprised parameters reported in literature (i.e. Arnold et al., 2012b, Me et al., 2015, Haas et al., 2015, Yen et al., 2014, Cerro et al., 2012, Omani et al., 2012, Chu et al., 2004). Parameters were sampled in a Latin hypercube sampling scheme of 1000 model runs. The objective function was the root mean square error of the simulations divided by the standard deviation of the observations (RSR; Moriasi et al., 2007b). The global parameter sensitivity was measured by the value of the *t*-test (and associated probability level *p*-value) of the regression coefficient of each parameter against the objective function, as well as using the visual appraisal of dot-plots (parameter values vs RSR). The analysis was conducted for each water management region, where most data was available, i.e. Austrian Danube, Morava, Vah-Hron-Ipel, Pannonian Danube, Drava, Sava and Tisa.

### 2.5 Assessment of model performance and analysis

To assess the model performances, for calibration and validation periods, both statistical and graphical techniques were used. The percent bias (PBIAS) was used as performance measure with reference to its corresponding class of performance (“very good”, “good”, “satisfactory” and “unsatisfactory”) based on recommendations of Moriasi et al. (2007b). The PBIAS measures the tendency of the simulated data to be higher or lower than the observations. Values close to 0 indicates a lack of bias (neither underestimation nor overestimation). Positive and negative values indicate an overestimation and underestimation of the simulated data, respectively. In this study PBIAS values in the range of ± 25% for monthly streamflow and ± 70% for monthly nutrient concentrations and loads were considered acceptable (Moriasi et al., 2007b). However, each class of performance was evaluated. The percent bias was calculated using the R package “hydroGOF” (Zambrano-Bigiarini, 2013).

Box and whisker plots, visual appraisal of time-series, and residuals analysis (observation -simulation) for each water management region were also performed (Harmel et al., 2014, Bieger et al., 2012). In addition, the simulated loads were assessed also considering the specific loads (total loads divided by total drained area, ton/km^2^/y).

## 3 Results and discussion

### 3.1 Calibration and validation of the model

#### 3.1.1 Crop yields

As a result of the calibration, the default base growth temperature of each crop (T_BASE) was set to the minimum value. The optimal growth temperature (T_OPT) was kept as default except for apple for which it was decreased from 20 °C to 18 °C. The harvest index (HVSTI) was also adjusted to match better the observed yields: it was increased for permanent crops (apple and vineyard), corn silage, durum and spring wheat, green beans, oats, sugar beet, sorghum hay and sunflower, while it was decreased for barley and potatoes.

**Fig. 2:**
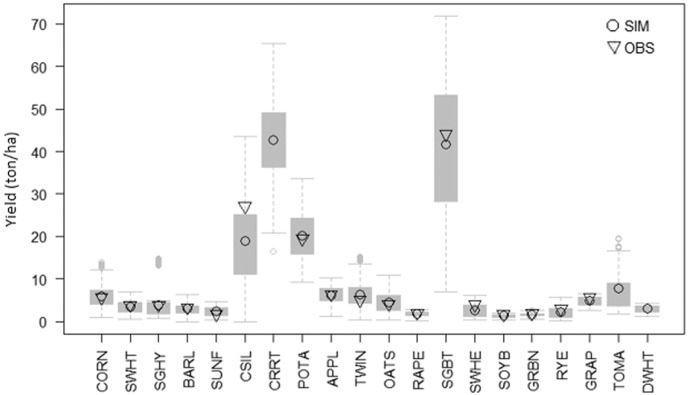
Box-and-whisker plots of SWAT annual crop yields of 20 simulated crops in the Danube River Basin. Crops order (left to right) is based on decreasing land extent in the Basin. Mean crop yield for 1995–2009 as observed (triangles) and simulated (circles) are reported. (CORN: corn; SWHT: Spring Wheat; SGHY: Sorghum Hay; BARL: Spring Barley; SUNF: Sunflower; CSIL: Corn Silage; CRRT: Carrot; POTA: Potato; OATS: Oats; SGBT: Sugar Beet; SOYB: Soybean; GRBN: Green Beans; RYE: Rye; GRAP: Vineyard; TOMA: Tomato; DWHT: Durum Wheat).

The comparison between the mean annual observed and simulated crop yields for the period 1995–2009 is shown in Fig. 2. The predicted mean annual crop yields compared well with the reported values, except for corn silage and sugar beet for which a slight underestimation can be noticed.

The annual variability of yields of dominant crops corn and spring wheat was well captured in all countries across the Danube, except in Austria (10% of the Basin), Ukraine (4% of the Basin), Slovenia (2% of the Basin) and Moldova (2% of the Basin) probably due to the misrepresenting of agricultural practices.

However, in the light of the simplification applied in the model setup and the data available, the crop yield calibration was considered satisfactory.

#### 3.1.2 Calibration and validation of streamflow

For the calibration of streamflow 26 parameters were independently changed at 264 gauged stations following the step-wise approach. Parameter sets of these stations were transposed in the different hydrological regions. Four hydrological regions were defined based on hydrological similarity (Malagó et al., 2015). In particular, the timing of peak flow was adjusted changing the snow parameters: the mean value of snowfall and snow melt temperature were set to − 0.84 °C and 0.60 °C, respectively; the decrease of temperature with elevation was estimated to be − 5.37 °C/km on average in the Basin. The snow melt rates (SMFMN and SMFMX) were both 5 mm H_2_O/day °C on average. The calibration of surface runoff resulted in an increase of the curve number (CN2) by about 10%, (except for artificially tile drained subbasins), and in a decrease of the Manning's value for tributary channel of 0.096 on average in the whole Danube. The lateral flow calibration was particularly sensitive to the soil available water content (SOL_AWC) and saturated hydraulic conductivity (SOL_K). Specifically, across the Danube Basin the SOL_AWC was increased by 10% on average, while the hydraulic conductivity by 16%. The aquifer percolation coefficient (RCHRG_DP) and the baseflow recession factor (ALPHA_BF) strongly influenced baseflow calculations across the Basin and their values changed substantially from the Upper to the Lower Danube. In the artificially tile drained subbasins, the depth of the impervious layer (DEP_IMP) was very sensitive and resulted 3100 mm on average, the depth of the subsurface tile (DDRAIN) resulted 690 mm, and the calibrated drainage lag time (GDRAIN) was 20 h on average in the whole Danube. The range of variation of all calibrated parameters is reported in Table 4.

Based on this final parametrization, the performance of SWAT model in simulating streamflow resulted satisfactory both for calibration and validation (Table 5). Specifically, the PBIAS% was acceptable (PBIAS ≤ ± 25%) for 70% and 61% of gauging stations in calibration and validation, respectively, resulting good to very good for 48% and 44% in the two datasets. The Interquartile Range (IQR) of monthly streamflow showed good agreement between SWAT simulations and observations (Table 6). The distribution of residuals (observations-simulations) was centred on zero and the IQR of residuals was [− 1.09; 5.05] m^3^/s in calibration and [− 2.64; 94.36] m^3^/s in validation with an increase of errors with the drainage area (see Supplementary material for graphical details, Fig. S3). Visual appraisal of monthly streamflow confirmed that monthly variations were well captured (see Supplementary material, Fig. S3).

**Table 5:** Overview of calibration, validation, and evaluation datasets with percentage (%) of gauging stations that performed according to Moriasi et al. (2007b) PBIAS model performance classes. The symbol # represents the number of gauging stations.

Dataset^a^	Data Type	# gauging stations	# data entries (period)	PBIAS performance class			
				Very good	Good	Satisfactory	Unsatisfactory
				(% of gauging stations)			
C	Q (m^3^/s)	264	37,074 (1995–2006)	36	12	22	30
V		708	126,375 (1995–2009)	33	11	17	39
C	N-NO_3_ (mg/L)	340	36,120 (1995–2009)	23	17	26	34
E	N-NO_3_ (ton/month)	202	21,666 (1995–2009)	26	15	33	27
C	TN (mg/L)	191	34,380 (1995–2009)	38	12	24	27
E	TN (ton/month)	121	5825 (1995–2009)	35	11	26	28
C	TP (mg/L)	333	59,940 (1995–2009)	25	11	12	52
E	TP (ton/month)	202	21,094 (1995–2009)	22	16	22	41

a C = calibration; V = Validation; E = evaluation.

**Table 6:** Overview of the main statistics of observed, simulated (SWAT) and residuals of monthly streamflow (m^3^/s), concentrations (mg/L) and loads (ton/month) of nutrients in each dataset.

Dataset^a^	Data type	# data entries		Percentiles				Mean
				25th	50th	75th	95th	
C	Q (m^3^/s)	37,074	Observed	1.35	2.92	6.19	18.01	5.38
			SWAT	1.02	2.88	6.41	19.05	5.41
			Residuals	− 1.09	0.13	1.15	5.05	− 0.02
V	Q (m^3^/s)	126,375	Observed	2.3	7.58	36.84	1059.84	198.54
			SWAT	2.18	8.08	38.38	912.53	190.21
			Residuals	− 2.64	0.16	3.73	94.36	8.32
C	N-NO_3_ (mg/L)	36,120	Observed	0.93	1.6	2.59	4.99	2.02
			SWAT	0.65	1.26	2.43	10.39	2.56
			Residuals	− 0.71	0.15	1.09	3.05	− 0.38
E	N-NO_3_ (ton/month)	21,666	Observed	11.3	49.33	354.6	11,773	1745
			SWAT	5.24	27.61	255.10	11,060	1858
			Residuals	− 11.8	5.4	54.89	1348	− 61
C	TN (mg/L)	34,380	Observed	1.77	2.62	3.93	10.60	3.72
			SWAT	1.29	2.39	3.86	14.6	3.91
			Residuals	− 1.20	0.02	1.28	5.94	− 0.48
E	TN (ton/month)	5825	Observed	19.0	134	1157	19,133	3144
			SWAT	6.2	48	534	17,051	2436
			Residuals	− 80	3.10	87	2482	− 9.05
C	TP (mg/L)	59,940	Observed	0.04	0.11	0.23	0.99	0.24
			SWAT	0.04	0.10	0.23	1.09	0.25
			Residuals	− 0.07	− 0.009	0.06	0.66	0.002
E	TP (ton/month)	21,094	Observed	0.57	2.87	24.67	734	123
			SWAT	0.43	2.64	26.08	581	109
			Residuals	− 3.04	− 0.03	1.97	161	23.41

a C = calibration, V = Validation; E = evaluation

#### 3.1.3 Nitrate-nitrogen calibration and evaluation

The calibration of the nitrogen transformation processes resulted in a nitrogen percolation coefficient (NPERCO) of 0.5, a denitrification exponential rate coefficient (CDN) of 2.5 and a denitrification threshold water content (SDNCO) of 1 (Table 4). The half-life parameter of nitrate in the shallow aquifer (HLIFE_NGW) was calibrated in each subbasin with values in the range 0–200 day^− 1^, resulting in 116 day^− 1^ (mean value) for the entire Danube. The initial nitrate concentration in the shallow aquifer was increased by about 10% in all subbasins.

**Fig. 3:**
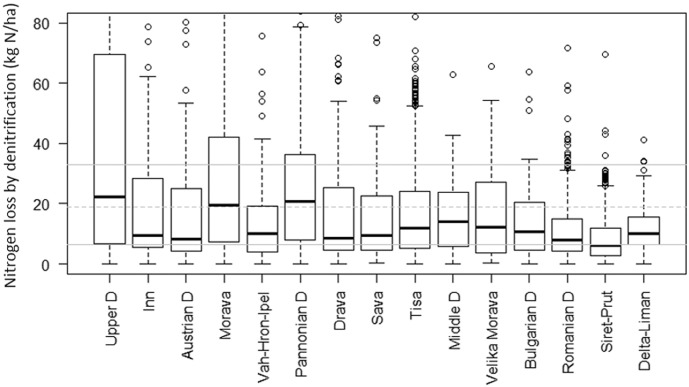
Box and whisker plots of mean annual denitrification (kg/ha) simulated in the period 1995–2009 in each ICPDR water management region. The grey dotted line indicates mean annual denitrification simulated in the whole Basin; the grey continuous lines indicate the 25th and 75th percentile of denitrification reported in literature (Velthof et al., 2009).

The mean annual denitrification was thus estimated for the whole Basin around 19 kg/ha (Fig. 3), very close to the median value of 21 kg/ha obtained from the MITERRA-EUROPE results (Velthof et al., 2009, Oenema et al., 2007). The denitrification decreased from Upper to Lower Danube reflecting the distribution of precipitation since lower precipitation leads to less soil saturation and thus less denitrification. The highest values of mean annual denitrification were observed in the Upper Danube (23 kg/ha), in the Morava (20 kg/ha) and in the Pannonian Danube (21 kg/ha), whereas the lowest values were generally simulated in the lower Danube, in particular in the Romanian Danube, Siret Prut and Delta Liman.

The soft calibration of denitrification led to satisfactory predictions of N-NO_3_ monthly concentration in 66% of gauging stations in the calibration dataset (Table 5). Mean SWAT N-NO_3_ concentration was 2.56 mg/L, which compares well with the observed mean of 2.02 mg/L (Table 6). The distribution of residuals was centred on zero with IQR of [− 0.71; 3.05] mg/L. Yet, the highest model residuals occurred at sites with smaller drainage areas, which are strongly affected by land management. Residuals decreased with increasing drainage area, since large basins better represent the “average” land management assumed in the model.

Better performances were obtained in the evaluation dataset. The PBIAS% calculated between the observed and simulated monthly loads ranked from satisfactory to very good for about 73% of total gauging stations (Table 5). The IQR of simulated monthly loads [10.4; 1177] ton/month were comparable to that calculated [5.24; 11,060] ton/month, albeit the 50th percentile was lower (27.6 ton/month) than that of observations (49.33 ton/month). The median value of monthly loads residuals was 5.4 ton/month, i.e. about 10% of median observation (Table 6). These deviations may partially be explained by the uncertainty related to the calculation of loads (Lloyd et al., 2016).

**Fig. 4:**
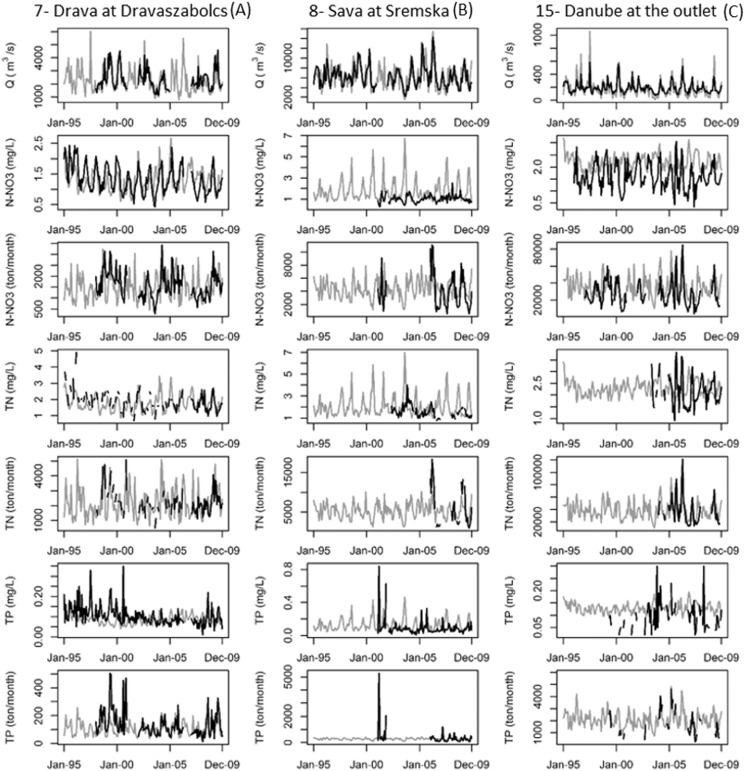
Monthly time series of streamflow (Q), nitrogen nitrate (N-NO3), total nitrogen (TN) and phosphorus (TP) concentrations and monthly loads observed (black line) and simulated by SWAT (grey line) at selected gauging stations across the Danube River Basin. The numbers in the title indicate the ICPDR water management region as reported in Table 1, whereas the letters identify their location in Fig. 1. Graphs for all water management regions in the Danube Basin are reported in the supplementary materials (Fig. S8–S14).

Simulated and observed monthly concentrations and loads for a sample of stations are shown in Fig. 4 (see also the Supplementary material).

#### 3.1.4 Total nitrogen calibration and evaluation

TN concentrations were calibrated in 191 gauging stations changing seven sensitive parameters (Table 4). First, the mineralization process was adjusted reducing the default values of the rate factor of humus mineralization of active organic nitrogen (CMN) to 0.000145 and the rate coefficient for mineralization of the residue fresh organic nutrients (RSDCO) to 0.2 in order to slow down the simulated kinetics; the N_UPDIS was increased from the default value (20) to 28 for better controlling the depth distribution of nitrogen uptake and thus the maximum amount of nitrate removed from the upper layers, as pointed out in Haas et al. (2015). The enrichment ratio of organic nitrogen (ERORGN) had a large impact on TN concentration and the median calibrated value was 0.7 for the whole Danube. The nitrogen settling rates in reservoirs were kept constant during the year and their range was set larger than the default values according to Panuska and Robertson (1999), as for instance in the Sava River Basin, to better simulate the significant retention of largest wetlands not implemented in SWAT (i.e. two Ramsar sites, three important bird areas and the alluvial wetlands in the Spacva –Bosut depression; Schneider-Jacoby, 2005).

As a result, the simulation of monthly TN concentration was satisfactory to very good for 74% of total gauging stations (Table 5). The IQR, median and mean of TN simulated concentrations were comparable to observed statistics (Table 6). The residuals were centred on zero, with median value of 0.02 mg/L and IQR of [− 1.20; 5.94] mg/L. However, as for N-NO_3_ case, residuals tended to decrease with increasing drainage area.

The monthly TN loads were satisfactory to very good for about 72% of gauging stations. The IQR of observed and simulated loads were comparable, albeit the median and mean differed slightly (Table 6), with SWAT simulations being lower than observed loads (Table 6). The distribution of residuals was centred on zero with median value of 3.1 ton/month and IQR of [− 80; + 87] ton/month.

Simulated and observed monthly TN concentration and loads are shown in Fig. 4 for a subset of stations: the Drava at Dravaszabolcs, Sava at Sremska Mitrovika and for the Danube Delta (see also the Supplementary material for other graphical comparisons).

#### 3.1.5 Total phosphorus calibration and evaluation

Monthly TP concentrations were calibrated adjusting the organic phosphorus enrichment ratio and the phosphorus settling rate in the reservoirs. The median value of these parameters was respectively of 0.1 and 57 m/year in the whole Danube.

TP simulation was satisfactory simulated up to very good for about 48% of calibrated gauging stations. The IQR of observed and simulated concentrations overlapped, with median of residuals well centred on zero (Table 6). These findings were confirmed in the evaluation, with about 60% of 202 the monitored stations reaching satisfactory results.

The highest percentage of unsatisfactory performance for TP in the calibration dataset likely reflects the difficulty of SWAT of reproducing high peak concentrations in events (Chu et al., 2004), which in turns may lead to errors in sediment flux estimates and particulate P (e.g. Me et al., 2015). The lower performance in simulating TP concentration may be linked to the local errors in simulating sediment fluxes as also observed in Vigiak et al. (2017).

### 3.2 Analysis of the water and nutrients fluxes per water management region

**Fig. 5:**
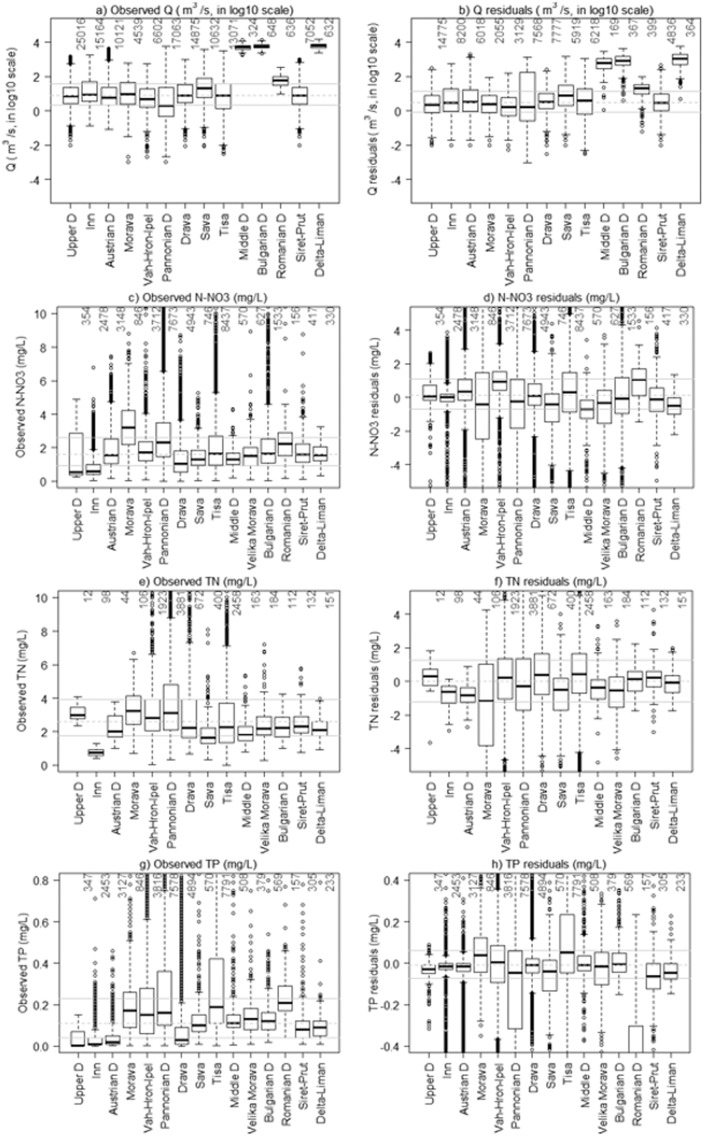
Left: distribution of observed values of streamflow and nutrients concentrations in each water management region. Right: SWAT simulations of residuals (observed–simulation). The grey continuous lines indicate the median value of the whole dataset, the grey dotted lines indicate the interquartile range. The numbers of data entries are reported above each box plot.

The mean observed average discharge of the Danube reaches approximately 2000 m^3^/s at the gauge Bratislava, 5500 m^3^/s at the gauge Iron Gate, and 6500 m^3^/s at the Danube Delta at the Black Sea. The main tributaries with the highest mean annual runoff are the rivers Inn in the Upper Danube section, and Sava and Tisa in the Middle Danube, leading to a significant increase of the mean annual streamflow of the Danube at their confluences. The analysis of residuals (observed-simulated) for each water management region allowed the identification of local errors across the Danube. Streamflow residuals increased with drainage area, with the highest residuals observed in the Middle Danube, Bulgarian Danube and Delta-Liman (Fig. 5a and b).

Observed monthly N-NO_3_ concentrations did not vary considerably between management regions (Fig. 5c). The long term average N-NO_3_ concentrations in the period 1995–2009 reached the highest values in the Morava River Basin (mean value around 3 mg/L) and Pannonian Danube region (mean value 2.8 mg/L). N-NO_3_ is the main component of TN (about 70% of TN) and both N-NO_3_ and TN concentrations decrease with increasing river size. The largest IQR was predicted in the Morava and Pannonian Danube, with average values typically observed for intensively agricultural watersheds, while in the Danube Source and Inn River Basin the concentrations were lower. N-NO_3_ concentrations were generally well captured in all water management regions, albeit overestimations were observed in Morava, Pannonian, Middle Danube, Siret Prut and Delta (Fig. 5d), whereas underestimations were detected in the Austrian Danube, Vah-Hron-Ipel and Romanian Danube. Furthermore, SWAT could not simulate accurately the seasonal variation of N-NO3 in the Inn River Basin, in the Upper Danube, and in the area between the Middle Danube and Delta-Liman. These findings highlighted the need to improve the spatial representation of denitrification in large river basins where its reliability is limited by setting the parameters at watershed level. In fact, in the Upper Danube soil denitrification resulted excessive and the simulation of N-NO_3_ could be improved by decreasing the watershed parameter CDN from 2.5 to 0.6, whereas in the Middle and Lower Danube soil denitrification CDN could be increased to 3 (see VII section of the Supplementary material and Fig. S19). Furthermore, the in-stream denitrification process is not simulated by SWAT, limiting the accuracy of prediction. However, nitrate concentrations were well captured in Drava, Prut and Siret River Basins.

Observed TN concentrations were the highest in the Morava and Pannonian Danube, with median value of 3.20 and 2.38 mg/L, respectively. At the Delta-Liman the long term average of TN concentration was 2.3 mg/L. In the Upper Danube regional differences were noticeable, with lowest TN concentrations observed in the Inn region whereas in the Upper Danube and in the Morava they were among the highest of the Basin (Fig. 5e). Conversely, TN varied little in the Lower Danube (from Middle to Delta-Liman). SWAT model was able to capture well these differences (Fig. 5f), albeit underestimations were observed in regions with limited data (i.e. Morava). However, TN concentrations at the outlets of the water management regions were all satisfactory (see Supplementary material).

Conversely to N-NO_3_ and TN, TP concentrations differed between water management regions, with highest values in predominantly agricultural regions, i.e. in Morava, Vah-Hron-Ipel, Pannonian Danube and Tisa (Table 1, Fig. 5g). The residuals were centred on zero in all water management regions except in the Romanian Danube, in which the median value of observed TP was similar to Tisa, and in Pannonian Danube region where underestimations were more frequent. Here the particulate P seems to be an important component of the TP that is moved with sediments to river network (Vigiak et al., 2017).

**Fig. 6:**
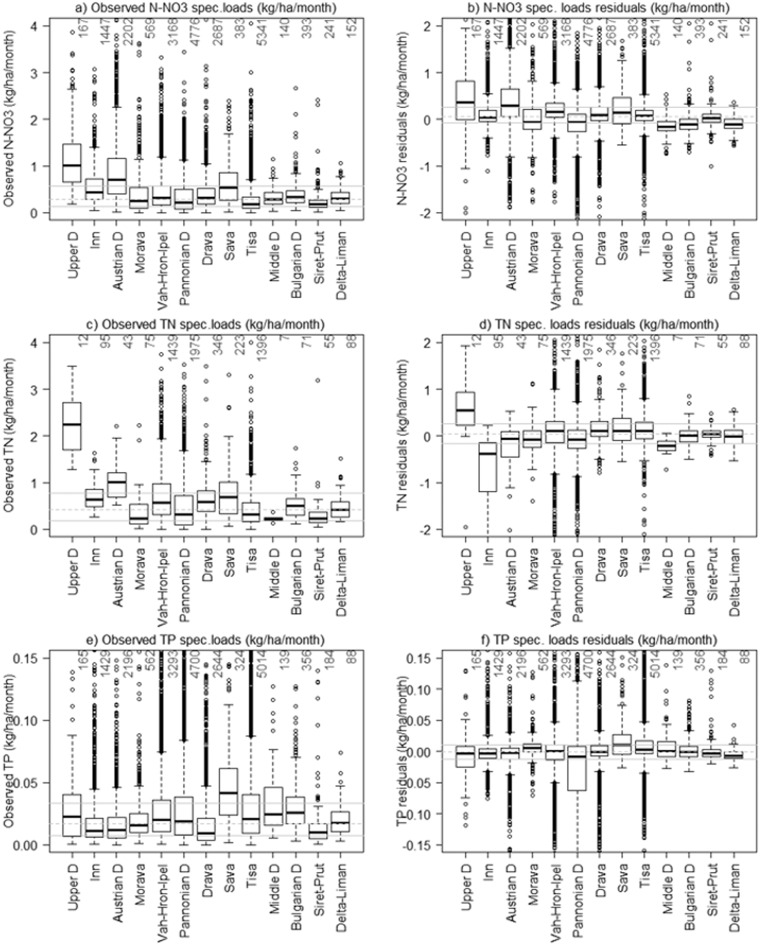
Left: distribution of observed values of nutrients loads in each water management region. Right: SWAT simulations of residuals (observed –simulation). The grey continuous lines indicate the median value of the whole dataset, the grey dotted lines indicate the interquartile range. The numbers of data entries are reported above each box plot.

The observed specific loads (loads divided by drainage area), of N-NO_3_ and TN vary considerably between the Upper Danube and other regions. Higher N-NO_3_ specific loads are observed in the Upper Danube and Austrian Danube (median value of 1 kg/ha/month and 0.6 kg/ha/month respectively), whereas in the other regions the median value is approximately 0.25 kg/ha/month (Fig. 6a). Fig. 6b clearly shows underestimations of specific loads in the Upper Danube and Austrian Danube, and slight overestimations in the Middle and Bulgarian Danube, as well as in the Danube Delta-Liman. In all other regions, the residuals were centred on zero.

The highest TN specific loads were observed in the Upper Danube and Austrian Danube with respectively median value of 2.4 kg/ha/month and 0.1 kg/ha/month, whereas the lowest values were in the Morava, Middle Danube and Siret-Prut regions. In the other regions, the median value was in the range of [0.3; 0.6] kg/ha/month. This variability was well captured by SWAT since the residuals were centred on zero in all water management regions (Fig. 6c and d), except in the Upper Danube and Inn where the specific loads were respectively underestimated and overestimated.

The TP specific loads were less variable than TN albeit high values were observed in the Sava River Basin (median value of about 0.3 kg/ha/month). The SWAT simulations matched well the observations and the residuals were all centred on zero (Fig. 6e and f). Yet, in the Pannonian region the underestimations of specific loads were more frequent as seen also for concentrations.

### 3.3 Analysis of the water and nutrients fluxes along the Danube River

**Fig. 7:**
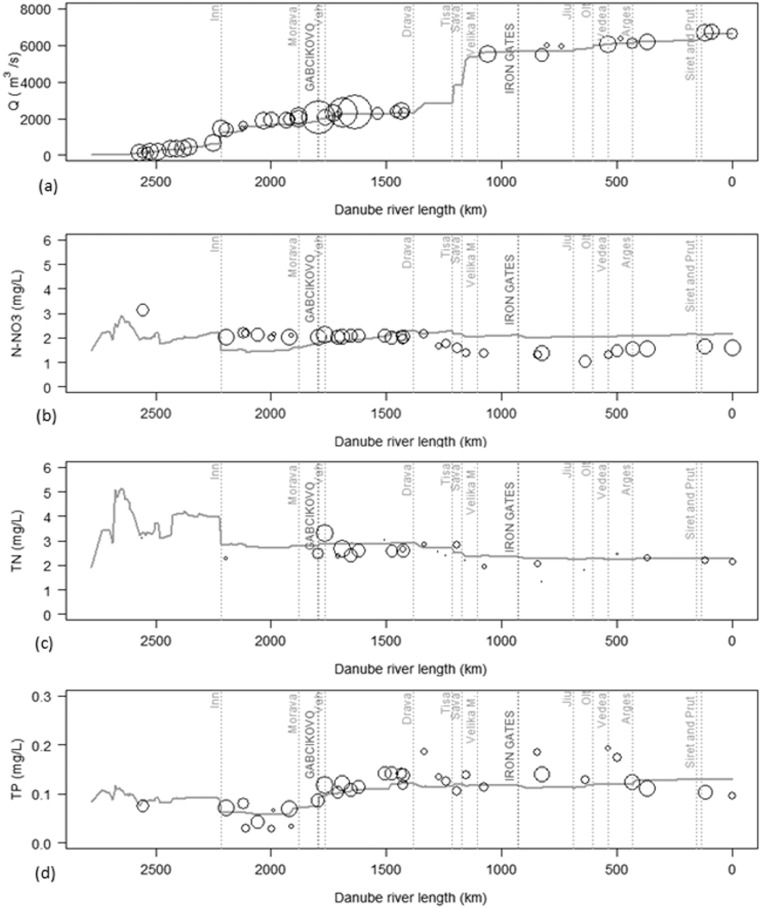
Long term average of monthly streamflow (a) and nutrient concentrations (b, c, d) along the Danube River with SWAT for the period 1995–2009 together with the available observations recorded at the gauging stations. The observations are represented with bubbles with size proportional to the number of total observations in the simulated period (1995–2009). The confluences of main tributaries, as well as the main barriers are indicated.

Fig. 7 shows the river Danube longitudinal profile of long term average monthly streamflow and in stream nutrients concentrations together with the observations. The observations are represented using bubbles with size proportional to the number of total observations in the simulated period (1995–2009), thus larger the bubble, the more observations were available for that station.

The monthly streamflow of the Danube River was well simulated, and in particular the sudden changes in correspondence with the confluence of Inn, Sava and Tisa tributaries were well captured (Fig. 7a).

The SWAT predicted that N-NO_3_ concentrations decreased stepwise following the streamflow increase, while the observations are quite constant from the confluence with the Inn and Tisa Rivers with long term mean value of about 2 mg/L. Between the confluence of Inn and the Morava Rivers, the SWAT N-NO_3_ concentrations were generally below the observations, while they were above after the confluence with the Tisa (Fig. 7b). These results confirmed that SWAT could not capture soil denitrification across the Basin. The inadequacy of the SWAT model to simulate the spatial distribution of soil denitrification was also recently pointed out in Epelde et al. (2016).

TN and TP concentrations were close to the observations albeit TP observations were at times inconsistent along the profile (Fig. 7c and d). TN concentrations slightly decreased stepwise following the streamflow increase from the confluence with the Inn River (3 mg/L) up to the Delta (2.3 mg/L). Before the Inn confluence the simulated TN concentrations reached the highest values, but the lack of data prevented the comparison with the observations. The simulated TP concentrations slightly increased after the confluence with the Inn River up to the Drava tributary and then remained constant at 0.12 mg/L. TP concentrations usually agreed well with the observations, although observations appeared to be more variable than SWAT simulations along the profile. However, we must point out that the simulated TP concentrations appeared not to be impacted by the Iron Gates reservoirs as reported in other studies (ICPDR, 2004, Van Gils and Bendow, 2000).

### 3.4 The water and nutrient balances of the Danube River basin

**Fig. 8:**
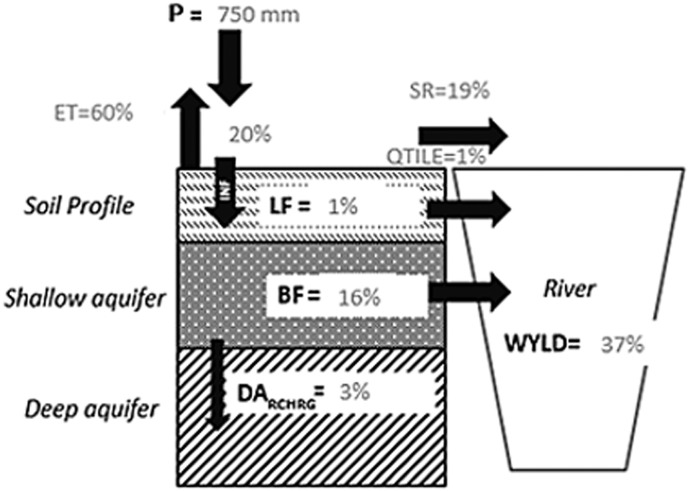
Long term water annual balance in the Danube River Basin according to SWAT model results in the period 1995–2009. P, precipitation; ET, evapotranspiration; BF, baseflow from shallow aquifer; INF, infiltration in the soil; LF, Lateral flow; SR, Surface Runoff; QTILE, tile drainage; DA_RCHRG_, the deep aquifer recharge; GW_RCHRG_, the shallow aquifer recharge; WYLD: water yield.

Fig. 8 shows the long term mean annual water balance for the entire Danube River Basin as simulated with SWAT for the period 1995–2009. For the Danube River Basin, it was estimated that 60% of the precipitation (P) was lost through evapotranspiration (ET) and 3% as percolation in the deep aquifer (DA_RCHRG_), and 37% was discharged in the stream (water yield, WYLD). Surface runoff (SR) and baseflow (BF) were respectively the main pathways of pollutants losses. The SWAT evapotranspiration and water yield results were very close to that reported in Petrovič et al. (2006) and EnviroGRIDS (2015), confirming the reliability of the model predictions.

**Fig. 9:**
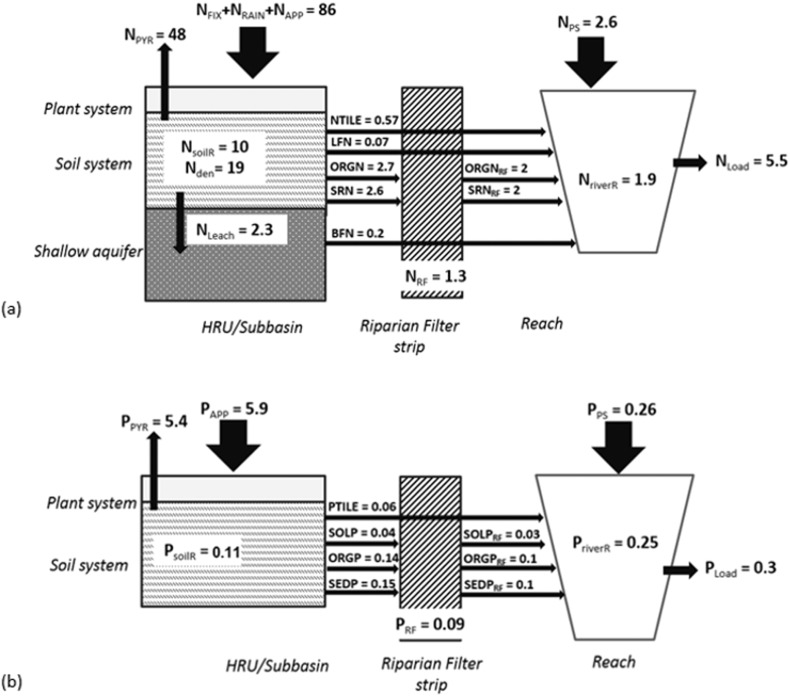
Long term mean annual nitrogen (a) and phosphorus (b) fluxes (kg/ha/year) in the Danube River Basin according to SWAT model results in the period 1995–2009. In (a): the diffuse sources are represented by the sum of nitrogen input via fixation (N_FIX_), nitrogen transported to the soil with the precipitation (N_RAIN_) and the nitrogen applied as fertilizer (N_APP_); N_PS_ is the nitrogen loading to the reach from point sources; the diffuse emissions are the nitrates loading to reach in tile drainage system (NTILE), in lateral flow (LFN), in surface runoff (SRN), in baseflow (BFN) and the organic nitrogen transported with the water yield (ORGN); ORGN_RF_ and SR_RF_ are respectively the organic nitrogen and nitrates reduced by riparian filtering; N_LEACH_ is the nitrogen leached to aquifer; N_PYR_, N_soilR_, N_aq_, N_RF_ and N_riverR_ are respectively the reduction of nitrogen applied by plant, soil, aquifer, riparian filter strip and river; N_Load_ is the total nitrogen load at the outlet of the Basin. In (b): the diffuse sources are represented by phosphorus applied as fertilizer (P_APP_); P_PS_ is the phosphorus loading to the reach from point sources; the diffuse emissions are the soluble phosphorus (phosphate) transported in tile drainage system (PTILE) and water yield (SOLP), the organic phosphorus loading to the reach (ORGP) and the mineral phosphorus adsorbed to sediment and transported into the reach (SEDP_RF_); SOLP_RF_, ORGN_RF_ and SEDP_RF_ are respectively the soluble, organic and mineral phosphorus reduced by riparian filtering; P_PYR_, P_soilR_, P_RF_ and P_riverR_ are respectively the reduction of phosphorus applied by plant, soil, riparian filter strip and river; P_Load_ is the total nitrogen loads at the outlet of the Basin.

Fig. 9a and b show the nitrogen and phosphorus fluxes and retentions in term of specific loads (kg/ha/year) in the Danube River Basin according to the SWAT model results. Regarding TN, the diffuse inputs were estimated at about 86 kg/ha, in which fertilizers application contributed 41.2 kg/ha, nitrogen from atmospheric deposition 13 kg/ha and the nitrogen fixed by plant 31.8 kg/ha. Point sources instead amounted to 2.6 kg/ha. The nitrogen removed by crop yield and lost in soils had the most significant impact on diffuse sources reduction with a reduction of 60% (48.2 kg/ha) and 37% (30 kg/ha), respectively. In particular, soil denitrification (constrained in the calibration procedure) was estimated to be around 19 kg/ha (24% of diffuse sources). Similar results were found in Pérez-Martin et al. (2016) who estimated a loss of annual average nitrogen surplus from soil of 26% in the Jùcar River Bain, Spain (43,000 km^2^) using PATRICAL model, whereas Hu et al. (2007) simulated a denitrification rate of 22 kgN/ha/year using SWAT in the upper Embarras River watershed, United States (482 km^2^). However, measurements indicate that the denitrification rates are highly variable in space and time, and are related to soil moisture, nitrate and carbon availability, and generally don't exceed 50 kg/ha (David et al., 2009). In addition, it was demonstrated that models, in which the denitrification process was not calibrated, predict long term average denitrification ranging from 3.8 to 21 kg/ha/year, with a larger intra-annual variability (David et al., 2009).

The nitrogen loss in the aquifer was estimated at 2.3 kg/ha (3% of diffuse sources reached the aquifers) and spatially decreases across the Danube Basin from the sources to the Delta (see the Supplementary material for the graphical detail). The total N emission, i.e. sum of nitrates loading to reach in tile drainage system, NTILE, nitrogen in lateral flow, LFN, in surface runoff, SRN, in baseflow, BF, and the organic nitrogen transported with the water yield, ORGN (see Fig. 9), was estimated of 6.14 kg/ha (corresponding to 492·10^3^ ton/year during the period 1995–2009, sum of NTILE, LFN, ORGN SRN and BFN in Fig. 9). SWAT emission estimates were comparable to MONERIS results (ICPDR, 2004) of about 7.8 kg/ha (corresponding to 623 · 10^3^ ton/year during the period 1998–2000) albeit loss pathways differed quite significantly. According to MONERIS, the main loss pathway was the groundwater (53%), whereas SWAT predicted most losses to occur via surface runoff (44%). Also the tile drain emission was different: for SWAT the tile drain contributed with 9% of total 492 · 10^3^ emissions in period 1995–2009 (44 · 10^2^ ton/year), while for MONERIS with 11% of total 623·10^3^ ton/year in the period 1998–2000 (about 68 · 10^2^ ton/year). The riparian filter strips reduced the diffuse emissions to 4.8 kg/ha, trapping about 20% of emissions from land. Furthermore, even though the point sources increased the loads in the river to 7.4 kg/ha, the in-stream retention amounted to about 30%, producing a final load of 5.5 kg/ha (corresponding to 459 · 10^3^ ton/year in the period 1995–2009).

**Fig. 10:**
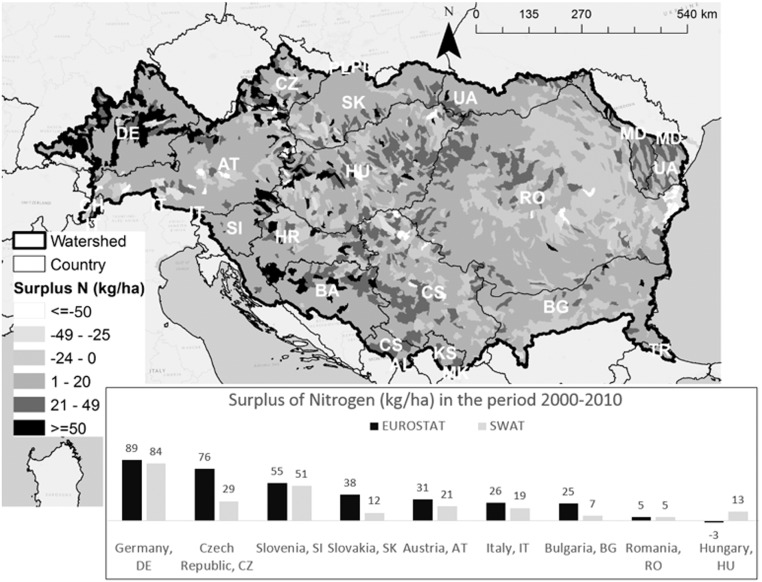
Map of nitrogen surplus in the period 2000–2010 calculated as difference between input (fertilization, nitrogen in rain and nitrogen fixation) and the uptake of plants and comparison of values by country between European statistics (EUROSTAT) and SWAT. The Countries shares following in the Danube Basin as follows: Germany, DE, 16%; Czech Republic, CZ, 28%; Slovenia, SI, 80%; Slovakia, SK, 97%; Austria, AT, 97%; Italy, IT, 19%; Bulgaria, BG, 56%; Romania, RO, 100%; Hungary, HU, 100%.

The spatial distribution of nitrogen surplus (the difference between nitrogen input through inorganic and organic fertilizer application, atmospheric deposition, fixation, and nitrogen uptake by crops), is shown in Fig. 10. Nitrogen surplus summarizes N potential losses from the subbasins during the period 2000–2010. The mean values at country level were compared to EUROSTAT gross nutrient surplus (EUROSTAT, 2012) and are shown in the same figure. It is noteworthy that nitrogen surplus was on average around 27 kg/ha in the whole Danube, with highest local values in the Upper Danube, Morava, Pannonian Danube and Sava Basin, where fertilizers application was significantly higher (see Supplementary material for details of amount of nutrients inputs by ICPDR regions). The SWAT nitrogen surplus at country level fit well the EUROSTAT estimations in particular in Romania, Austria, Slovenia and Germany. Some differences were noticeable in Slovakia where SWAT underestimated the nitrogen surplus; this may be due to simplifications in the manure fertilization set-up. In terms of TP, fertilizers application was estimated to be around 6 kg/ha, with plant uptake capturing 90% of TP. TP diffuse emissions were estimated at 0.38 kg/ha (i.e. 44 · 10^3^ ton/year for the period 1995–2009), lower than MONERIS estimate of 0.56 kg/ha (45 · 10^3^ ton/year during the period 1998–2000; ICPDR, 2004). The main emission pathways were related to the transport of organic phosphorus via surface runoff and lateral flow in accordance with other studies (Chardon et al., 1997, Turner and Haygarth, 2000) and to hillslope erosion, which is strongly linked to the catchment morphology, vegetation and land use (Steegen et al., 2001). The erosion was the main P pathway also for MONERIS (ICPDR, 2004). Dissolved phosphate transported via tile drains contributed only about 0.06 kg/ha. The SWAT TP diffuse emission (0.39 kg/ha) were further reduced by riparian filter strips to 0.3 kg/ha (about 13% of retention). With the addition of point sources load (0.26 kg/ha), TP loads reaching the stream network amounted to 0.55 kg/ha. However, in-stream retention was 50%, resulting in 0.3 kg/ha of TP loads in the river, corresponding to 25,173 ton/year in the period 1995–2009.

### 3.5 Usefulness of model predictions and future applications

The analysis of model predictions and limitations related to data availability and model structure, provides valuable insights on water and nutrient processes at different spatial (subbasin, river, basin) and temporal scales (monthly and annual). This “state-of-the-art” modelling of water quantity and quality of the Danube Basin identified critical spots where additional in-depth investigations may be necessary. For instance, to better support catchment management the monitoring network of streamflow should be strengthened in the Velika Morava, Middle Danube, Romanian and Bulgarian Danube, whereas harmonized monitoring of nutrient concentrations should increase in the Upper Danube, Sava River Basin and in all regions from Middle Danube to Delta (Chapman et al., 2016).

Predicting nutrient concentrations rather than loads, as shown in this study, offers several advantages. First, it avoids the uncertainty issues related to loads estimation. Second, it supports the implementation of the EU environmental Directives that set targets in terms of nitrogen concentration. For example, it can help to control the maximum allowable NO3 concentration of 50 mg/L according to the Drinking Water Directive (98/83/EC) and identify sites where mean annual NO_3_ concentrations exceed 50 mg/L as required by the Nitrates Directive (91/676/EEC). Third, predicting concentrations provides information that can be directly used in the definition of the risk assessment, establishing concentration-effect relationships for the aquatic ecosystems.

**Fig. 11:**
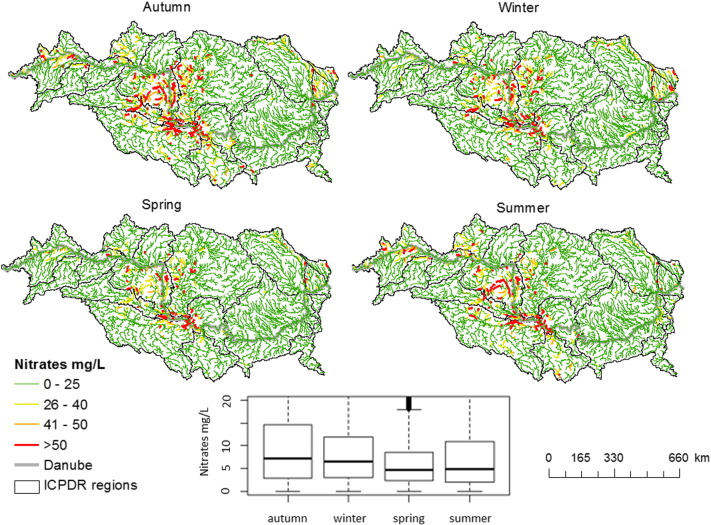
Maps and Box and whisker plot of long term mean seasonal nitrate concentration (NO_3_ mg/L) according to SWAT model results in the period 1995–2009. The nitrate concentration was calculated from the simulated SWAT N-NO_3_ using a conversion factor of 4.427.

For a direct comparison with the maximum contaminant concentration of nitrate (50 mg/L NO_3_) for potable water according to the World Health Organization (WHO), Fig. 11 shows the spatial distribution of long term mean monthly nitrate concentration calculated from the simulated N-NO_3_ (conversion factor of 4.427). The figure shows in detail the variation of nitrate concentration in rivers in the four seasons, from autumn to summer, for the period 1995–2009. The highest nitrate concentrations occur during autumn and winter with median values of 7.3 and 6.5 mg/L respectively (IQR ranges of [2.8; 14.5] mg/L and [3; 11.8] mg/L respectively), while they are lower during summer (median of 5 mg/L; IQR range [2; 10] mg/L) and spring (median 4.5 mg/L; IQR range [2.3; 8.6] mg/L). These values are correlated to the nitrogen surplus in soils and the dilution effect of stream flow, more pronounced during the spring season. Nitrate seasonal concentrations are also correlated to the magnitude of the denitrification process that occurred when soils are saturated and in an anaerobic environment. This process is heavily influenced by temperature as well. During autumn and winter the rate of denitrification is greatly reduced due to the lower temperature, while during spring and summer the environmental conditions are more favourable to microbial activity. However, even during summer, some rivers in the Upper Danube, Pannonian Sava and Middle Danube exceed the 50 mg/L of nitrate.

Spatially NO_3_ concentrations exceed 50 mg/L in the Pannonian Danube, Sava at the outlet and at the entrance into the Middle Danube during all seasons with some peaks of concentrations also in the Upper Danube during autumn and summer (Fig. 10). This distribution can be explained by the highest percentage of cropland and thus nitrogen inputs in the Pannonian Danube.

Besides understanding the processes and pathways responsible for water nutrient pollution, and identifying problematic areas where actions should be targeted, the modelling approach presented in this study can support the analysis of scenario, as internal feedbacks between water and nutrient cycles are considered, as well as the effects of crop growth and land and water management. Having predicted the water, nutrients and sediments (Vigiak et al., 2017) fluxes in current conditions, the next steps are to assess the impact of management scenarios on ecosystem services, evaluate best management practices on the ecological status of the river (Grizzetti et al., 2016, Vigiak et al., 2016), search for economically effective solutions, and identify trade-offs between economics and pollution status (Udias et al., 2016). Finally, the Danube Basin SWAT model results could support the development of indicators of water, energy and food securities in a “Nexus Thinking” approach with reference to the Sustainable Development Goals of the United Nations (Giupponi and Gain, 2016).

### 3.6 Consideration about the nutrient pollution in the Danube River Basin and the end up in the Black Sea

The Danube River and its catchment provide many services including drinking, industrial and agricultural water supply, hydroelectric power generation, navigation, tourism, recreational opportunities and fisheries. These intensive uses have created severe pressures on water quality and quantity affecting biodiversity in the Basin, and polluting large areas of the Black Sea (Popovici, 2014).

This study of nutrient balances and the seasonal analysis of nitrates has allowed identifying the impact of diffuse and point sources on the Danube River and its tributaries. Elevated loads of nitrogen enter the river through diffuse sources such as fertilization. In particular, the use of mineral fertilizers significantly contributes to the pollution of the Danube River (55%), followed by manure (37%) and ammonia (8%). However, the agricultural impact is substantially reduced by around 90% mainly due to crop uptake, soil denitrification and riparian filter strips (reduction of 60%, 24% and 2%, respectively). The nitrogen from urban settlements directly contribute to the load of the Danube River by about 35%, while the diffuse emissions from agricultural sector (including the contribution from precipitation) account for 65%.

Similarly, high phosphorus loads enter in the Danube Basin throughout fertilizer application (95% of total sources). However, this potential impact is substantially reduced by plant uptake and only 5% of diffuse sources reach the river system that is heavily stressed by the phosphorus pollution from point sources.

The Black Sea, which ultimately receives the waters of the Danube Basin, is sensitive to eutrophication and the severe eutrophic conditions of the late 1980s might occur again if agriculture and waste water treatment plants discharges are not managed properly.

**Fig. 12:**
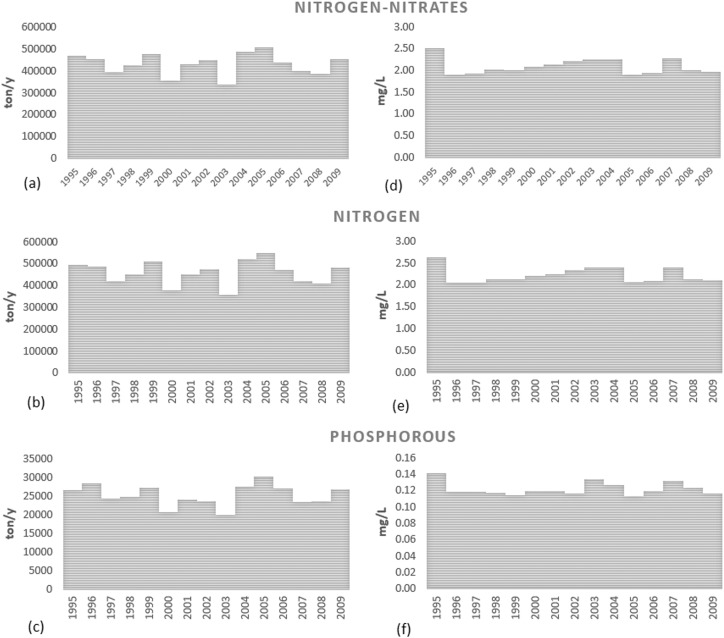
Annual loads (a, b, c) and concentrations (d, e, f) of nitrogen-nitrates, total nitrogen and phosphorus discharge into the Black Sea.

Fig. 12 shows the annual variation of nutrient loads and concentration entering into Black sea in the period 1995–2009 as simulated by the SWAT model. The Danube on average introduces about 25,000 tonnes of P, 460,000 tonnes of N and 432,000 of N-NO_3_ into the Black Sea each year. The concentrations of N-NO_3_, N and P are quite constant in the period 1995–2009 with average values of 2.1, 2.2 and 0.12 mg/L, respectively.

The mean annual specific nutrient fluxes exported from the Danube Basin to the Black Sea were also compared with the observed riverine fluxes reported in Billen et al. (2011). The SWAT nitrate-nitrogen and total nitrogen fluxes exported to the Black sea in the period 1995–2009, estimated around 540 and 570 kg/km^2^/year respectively, resulted similar to that predicted by MONERIS model for the 2001–2005 period (560 kg/km^2^/year) but lower than the estimation of Cociasu et al. (1996) of 860 kg/km^2^/year for the period 1952–1992. This result confirms the efforts done to control nutrient enrichment over the past 30 years Popovici (2014). However, the modelled nutrient fluxes of the Danube resulted lower than other European basins with similar percentage of cultivated land. For instance, the Rhine River Basin, characterized by 50% of arable land, delivery at the outlet around 2000 kg/km^2^/year (Lancelot et al., 1991), and the Elbe (57% of arable land) discharges around 1000 kg/km^2^/year (Radach and Pätsch, 2007). The mean annual specific N-NO_3_ yield is also comparable with other basins in Northeastern US (Mayer et al., 2002), in Germany (Langusch and Matzner, 2002) and in Northern Taiwan (Kao et al., 2004) that are characterized by small or moderately agricultural extent. Concerning the phosphorus, the SWAT total phosphorus fluxes exported to the Black Sea in the period 1995–2009 was comparable to that estimated by Cociasu et al. (1996) around 30 kg P/km^2^/year. This value is lower than the delivery phosphorus fluxes from other European river basins such as Rhine (242 kg P/km^2^/year, Lancelot et al., 1991) and Rhone (78 kg P/km^2^/year; Rabouille et al., 2008).

## 4 Summary

A process-based modelling approach, that involves a systematic calibration/validation strategy of SWAT model, was developed and applied in the Danube River Basin to predict streamflow, sediments and nutrients fluxes at different spatial and temporal scale. The methodology involved the use of both soft data (i.e. literature information of denitrification, crop yields from statistics) and hard data (i.e. long time series of streamflow and concentrations). The calibration/validation of streamflow was developed in steps in order to simulate adequately each hydrological component, while sediment and nutrients were calibrated using directly the concentrations. The use of concentrations for model calibration enabled direct support for the implementation of EU environmental Directives.

The results show that SWAT is able to adequately represent crop yield, monthly streamflow, total nitrogen (TN) and total phosphorus (TP) concentrations in the Danube Basin, albeit the peaks of TP concentration were at times underestimated. Difficulties were encountered in reproducing the nitrogen-nitrate concentration seasonality across the Basin due to the simplification of SWAT model in representing soil denitrification process.

Model simulations were analysed per water management regions identifying areas with important knowledge gaps where an in-depth analysis may be necessary. Furthermore, the analysis of model results along the Danube River provided insightful information on the tributaries impact on Danube water quantity and quality.

The mean annual water balance has allowed identifying the main components that affect the water balance giving an overview of the actual water resources status. This “state-of-the-art” modelling of the water resources and nutrients pollution in the Danube River Basin offers an important step forward in large scale integrated modelling. Despite necessary simplifications, accurate results can be obtained while keeping realism in the representation of the physical processes and as such the model can be used to support effective water management.

## Appendix A Supplementary data

Supplementary material Image 1
