# Supplementary material for: Modelling water and nutrient fluxes in the Danube River Basin with SWAT
Source: Sci Total Environ. 2017 Dec 15;603-604:196–218. doi: 10.1016/j.scitotenv.2017.05.242 (PMC5535642; doi:10.1016/j.scitotenv.2017.05.242)
Supplement: Supplementary file 1 — Supplementary material [file mmc1.docx]

**Supplementary material**

**This supplementary material provides useful information about the SWAT model application of this study, as well as additional** visual appraisal of model results using both time series plots and maps.

- - 1. **SWAT model version**

In this study the executable SWAT2012 rev622 was ran and the ArcSWAT interface (ArcSWAT Version 2009.93.7b) was used to compile the SWAT input files.

In particular, the SWAT 2012 code v.622 was modified to create new outputs: the *subflow.rch* that contains the streamflow and its components (surface runoff, lateral flow, baseflow), as well as the logarithm of streamflow (in m^3^/s); the *sedflow.rch* improves the existing *output.sed* writing 26 variables that were originally subdivided in the *output.sed* and *output.rch* (one of the most important file of SWAT that writes the simulated streamflow output and related qualitative characteristics in each reach); the *qualflow.rch* is a new output file that writes the concentration (mg/l) of total nitrogen and phosphorus, as well as its chemical forms (Nitrate-N, N-NH3, Nitrite-N, N-NH2, Organic nitrogen ORGN, Ammonium-N, N-NH4, Phosphate-P, P-PO4 and Organic phosphorus, ORGP).

These files were compatible with SWAT-CUP software (Abbaspour et al, 2008), that was used in the calibration strategy.

- - 1. **SWAT model version**

The calibration and validation strategy applied follows a cascade approach as shows in S1.

- - 1. **The simulated annual crop yields by countries**

Figure S2 shows the comparison between annual observed and simulated crop yields (ton/ha) for corn and wheat in the period 1995-2009 and for each country across Danube. These crops cover the largest area in the crop land.

The figure shows that SWAT reproduces well the annual variability of crop yields, except in Slovenia and Ukraine Moldova and Austria where maybe different crops were cultivated and/or the management was not accurately simulated.

- - 1. **The model simulations against observations and relation with other variables**

Figure S3 show the comparison of SWAT monthly streamflow with observations in validation and calibration dataset, as well as in relation to drained area (km^2^). Instead the nutrients concentrations and loads were compared to observation and were related to streamflow (Figures S3, S4, S5, S6). It is noticeable that the monthly streamflow residuals increase when the drain area increases, whereas the residuals of concentrations and loads of nutrients were well centred on zero with large errors in correspondence of low streamflow values.

- - 1. **Maps and visual appraisal of model performances**

Figures S7 a,b,c,and d show the maps of streamflow and nutrients concentrations resulting from the SWAT model in the period 1995-2009 and the PBIAS values in each gauging station. This map was created directly using the outputs of SWAT and a script written in R software language (R Development Core Team, 2009).

Figures from S8 to S14 show instead the visual appraisal of model results using time series in each water management region.

Figure S15 shows the long term mean annual loads along the Danube. Generally, the loads increased stepwise following the streamflow changing suddenly in correspondence of tributaries, whereas the effect of main barriers had not markedly dropped the loads.

- - 1. **Spatial distribution of some nutrients balance components**

Figures from S16 to S18 show the spatial distribution of selected components of the nutrient balance: nitrogen leached in the aquifers, nitrogen-nitrates loading to the reach in surface runoff and the mineral phosphorus adsorbed to sediment and transported into the reach.

The main source of nutrient pollution for each ICPDR region were reported in Table S1.

- - 1. **The calibration of denitrification process**

Figure S19 show the spatial variation of long-term mean annual denitrification (kg/ha) in the Danube River Basin (period 1995-2009) with 9 combinations of the parameters CDN and SDNCO.

- a1) CDN=0.6 and SDNCO=1
- a2) CDN=0.6 and SDNCO=0.9
- a3) CDN=0.6 and SDNCO=0.8
- b1) CDN=1.4 and SDNCO=1
- b2) CDN=1.4 and SDNCO=0.9
- b3) CDN=1.4 and SDNCO=0.8
- c1) CDN=3 and SDNCO=1
- c2) CDN=3 and SDNCO=0.9
- c3) CDN=3 and SDNCO=0.8

Among the 9 configurations, a1, b1 and c1 were considered able to simulate a realistic denitrification in the Danube River Basin since saturation of soils was forced imposing SCDNO to 1. The others were instead unrealistic.

The long term mean annual denitrification increased with decreasing the SDNCO from 1 to 0.8, and with increasing the CDN. The highest value of denitrification (31.5 kg/ha) was obtained with CDN and SDNCO respectively equal to 3 and 0.8, while the lowest value (13.5 kg/ha) was simulated when CDN was 0.6 and the SDNCO equal to 1 (see Figure S20).

The configuration c1 was considered the most reliable to simulate the denitrification in the Danube in accordance with the annual value retrieved from literature. In this study, CDN was then slightly decrease to 2.5 to better simulate the monthly variability of nitrogen nitrates in the Upper Danube.


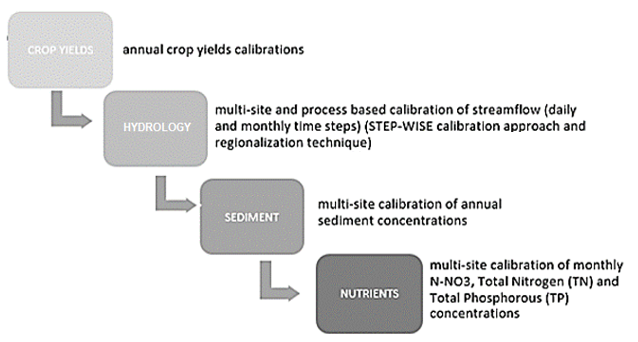


Figure S. 1. Strategic C/V approach using the SWAT model.


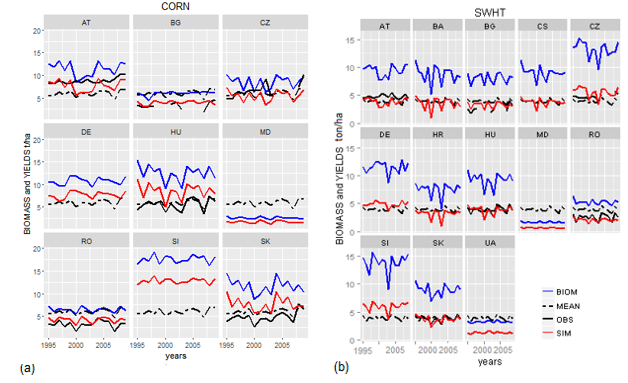


Figure S. 2. Comparison of annual observed (OBS, continuous black line) and simulated (SIM, continuous red line) crop yields (ton/ha) for the corn (CORN; a) and wheat (SWHT, b). The black dotted lines indicate the mean of annual observed values in the entire Danube, while the continuous blue lines indicate the biomass (ton/ha). AT: Austria; BA: Bosnia and Herzegovina; BG: Bulgaria; CS: Serbia and Montenegro; CZ: Czech Republic; DE: Germany; HR: Croatia; HU: Hungary; MD: Moldova; RO: Romania; SI: Slovenia; SK: Slovakia; UA: Ukraine.


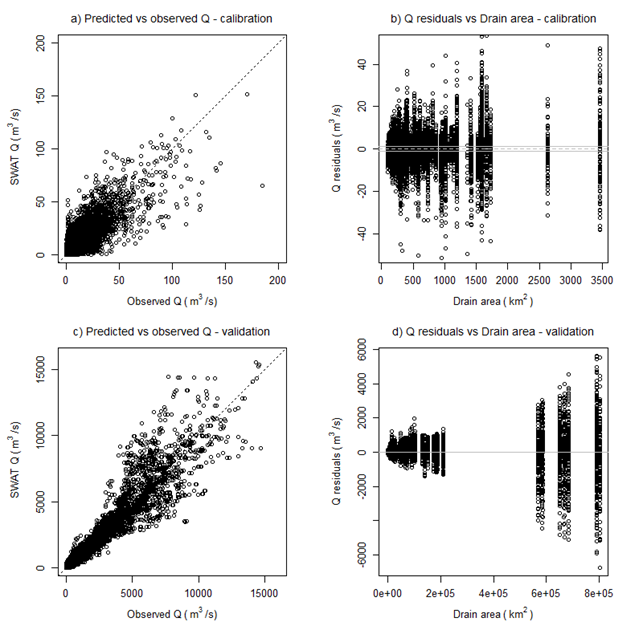


Figure S. 3. Comparison of SWAT monthly streamflow against observations. On the left scatterplots of predictions versus observation for the calibration (a) in the period 1995-2006 and validation (c) in the period 1995-2009; on the right the distribution of monthly streamflow residuals (observation-simulation, m^3^/s) in relation to drained area (km^2^). The grey dotted lines indicate median and interquartile of residuals.


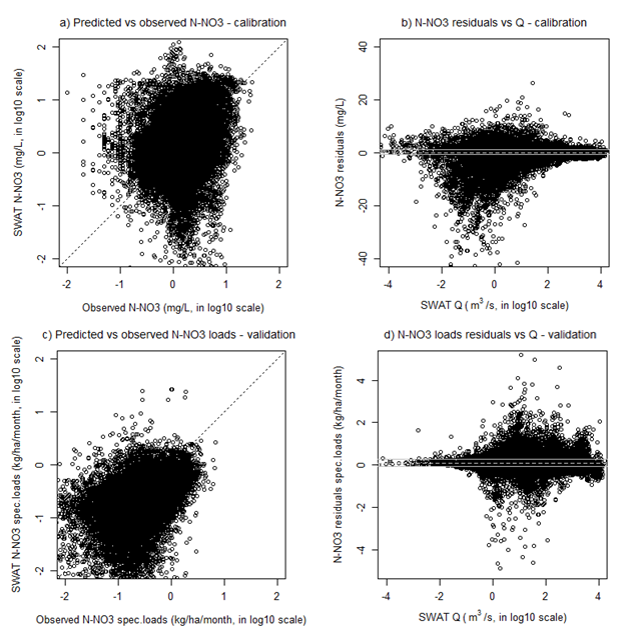


Figure S. 4. Comparison of SWAT monthly nitrogen nitrates against observations in the period 1995-2009. On the left scatterplots of predictions versus observation for the calibration (a) and validation (c) datasets in terms of concentration (mg/L, in in log 10 scale) and specific loads (kg/ha/month in log 10 scale) respectively. On the right the distribution of monthly nitrogen nitrates residuals (observation-simulation, m^3^/s) in relation to monthly simulated streamflow (m^3^/s in log10 scale) in the calibration (b) and validation (c) datasets. The grey dotted lines indicate median and interquartile of residuals.


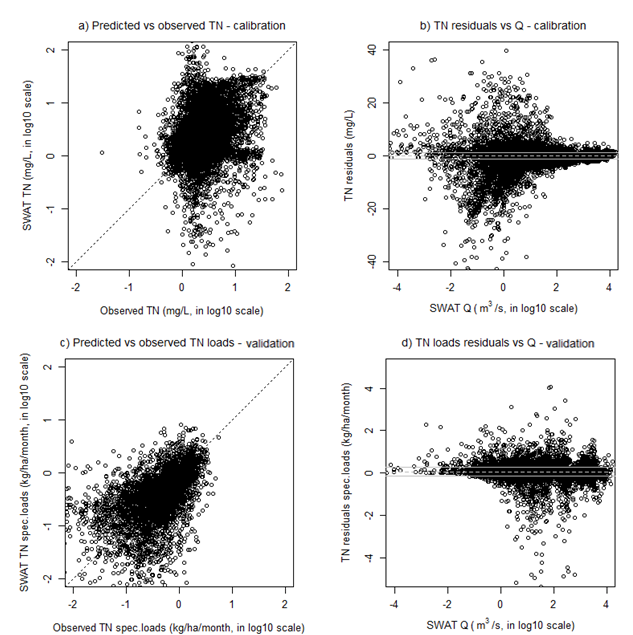


Figure S. 5. Comparison of SWAT monthly total nitrogen against observations in the period 1995-2009. On the left scatterplots of predictions versus observation for the calibration (a) and validation (c) datasets in terms of concentration (mg/L, in in log 10 scale) and specific loads (kg/ha/month in log 10 scale) respectively. On the right the distribution of monthly total nitrogen residuals (observation-simulation, m^3^/s) in relation to monthly simulated streamflow (m^3^/s in log10 scale) in the calibration (b) and validation (c) datasets. The grey dotted lines indicate median and interquartile of residuals.


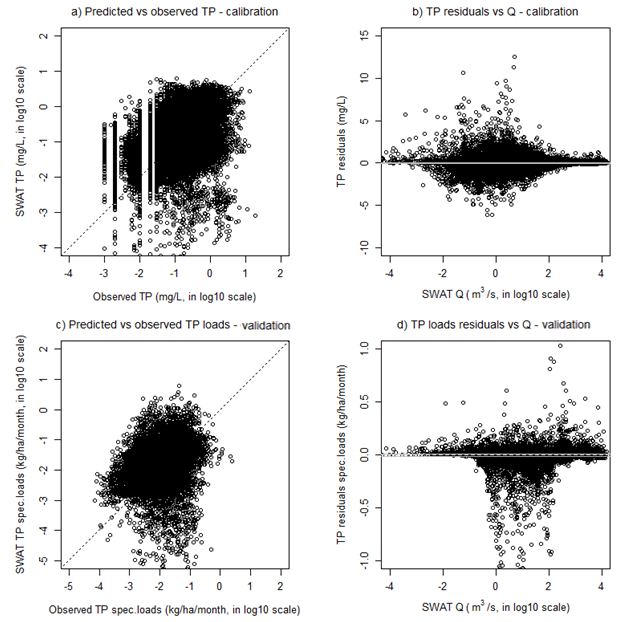


Figure S. 6. Comparison of SWAT monthly total phosphorus against observations in the period 1995-2009. On the left scatterplots of predictions versus observation for the calibration (a) and validation (c) datasets in terms of concentration (mg/L, in in log 10 scale) and specific loads (kg/ha/month in log 10 scale) respectively. On the right the distribution of monthly total phosphorus residuals (observation-simulation, m^3^/s) in relation to monthly simulated streamflow (m^3^/s in log10 scale) in the calibration (b) and validation (c) datasets. The grey dotted lines indicate median and interquartile of residuals.


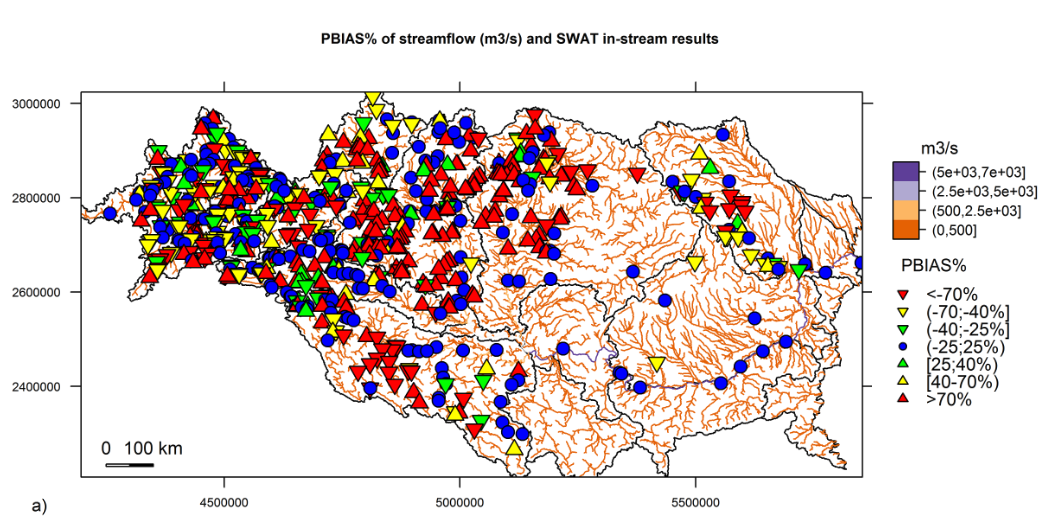


Figure S. 7a. Percent bias (PBIAS %) calculated between monthly simulated and observed stream flow Q (a) in the period 1995-2009; positive PBIAS% indicates overestimation, while negative PBIAS% indicates underestimation. In the reaches the SWAT long-term mean monthly values are showed. This maps were created with automatic script developed using the R software (R Development Core Team, 2009).


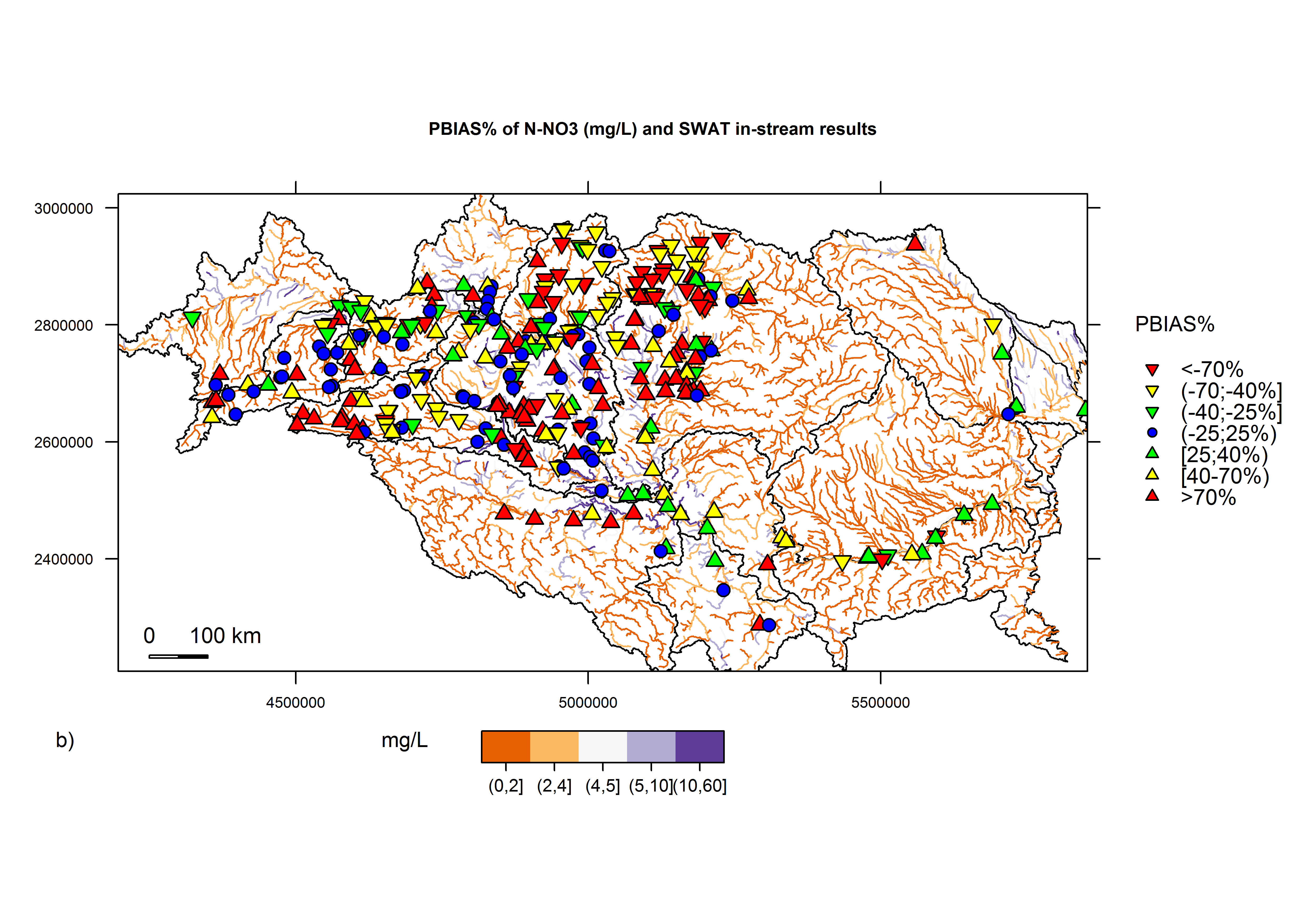


Figure S. 7b. Percent bias (PBIAS %) calculated between monthly simulated nitrate-nitrogen (b) in the period 1995-2009; positive PBIAS% indicates overestimation, while negative PBIAS% indicates underestimation. In the reaches the SWAT long-term mean monthly values are showed. This maps were created with automatic script developed using the R software (R Development Core Team, 2009).


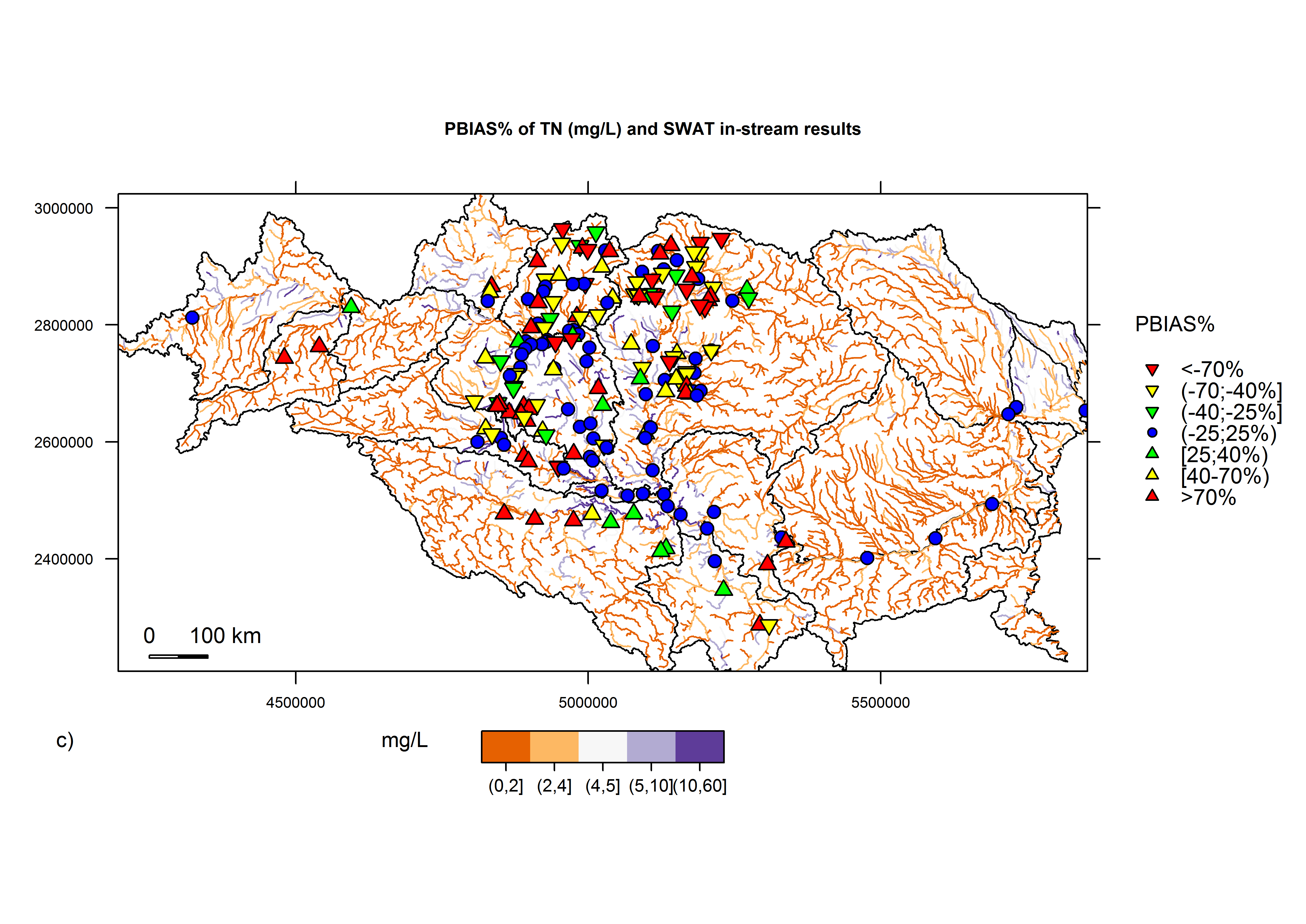


Figure S. 7c. Percent bias (PBIAS %) calculated between monthly simulated and observed total nitrogen (c) in the period 1995-2009; positive PBIAS% indicates overestimation, while negative PBIAS% indicates underestimation. In the reaches the SWAT long-term mean monthly values are showed. This maps were created with automatic script developed using the R software (R Development Core Team, 2009).


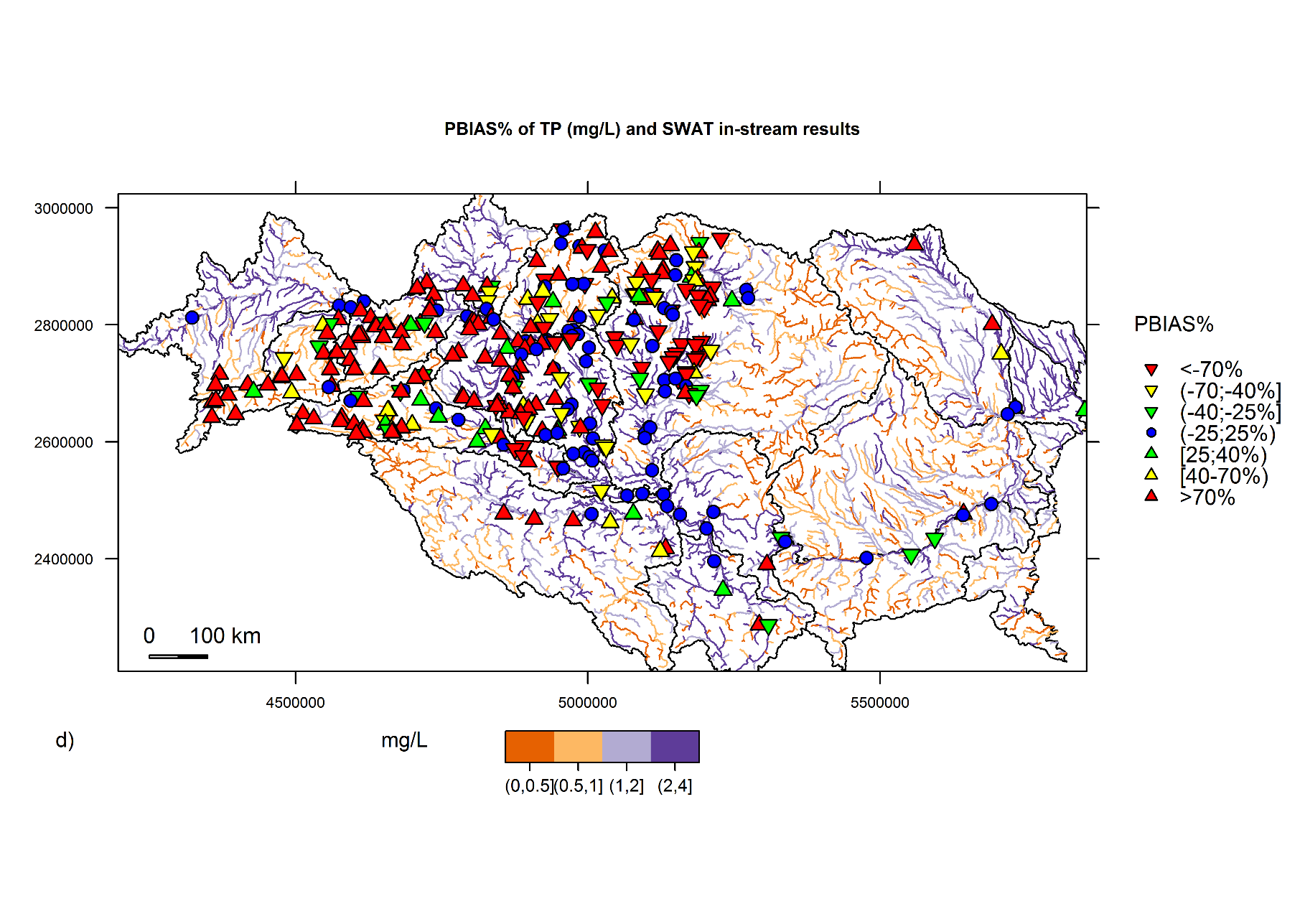


Figure S. 7d. Percent bias (PBIAS %) calculated between monthly simulated and observed total phosphorus (d) in the period 1995-2009; positive PBIAS% indicates overestimation, while negative PBIAS% indicates underestimation. In the reaches the SWAT long-term mean monthly values are showed. This maps were created with automatic script developed using the R software (R Development Core Team, 2009).


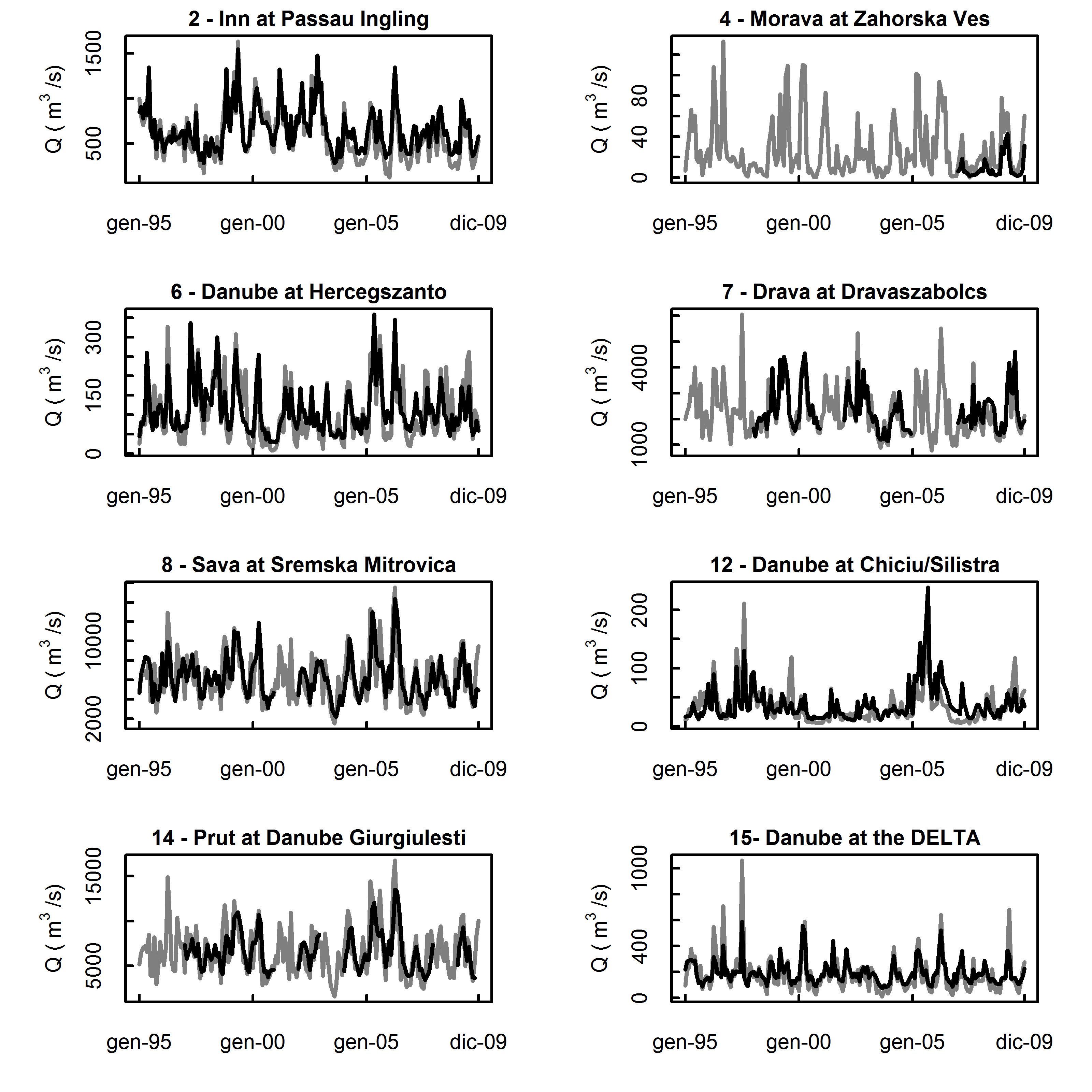


Figure S. 8. Monthly time series of streamflow Q (m^3^/s) as observed at the outlet of some water management regions (black line) and as simulated by SWAT model (grey line) for the period 1995-2009. Note the differences in y axis.


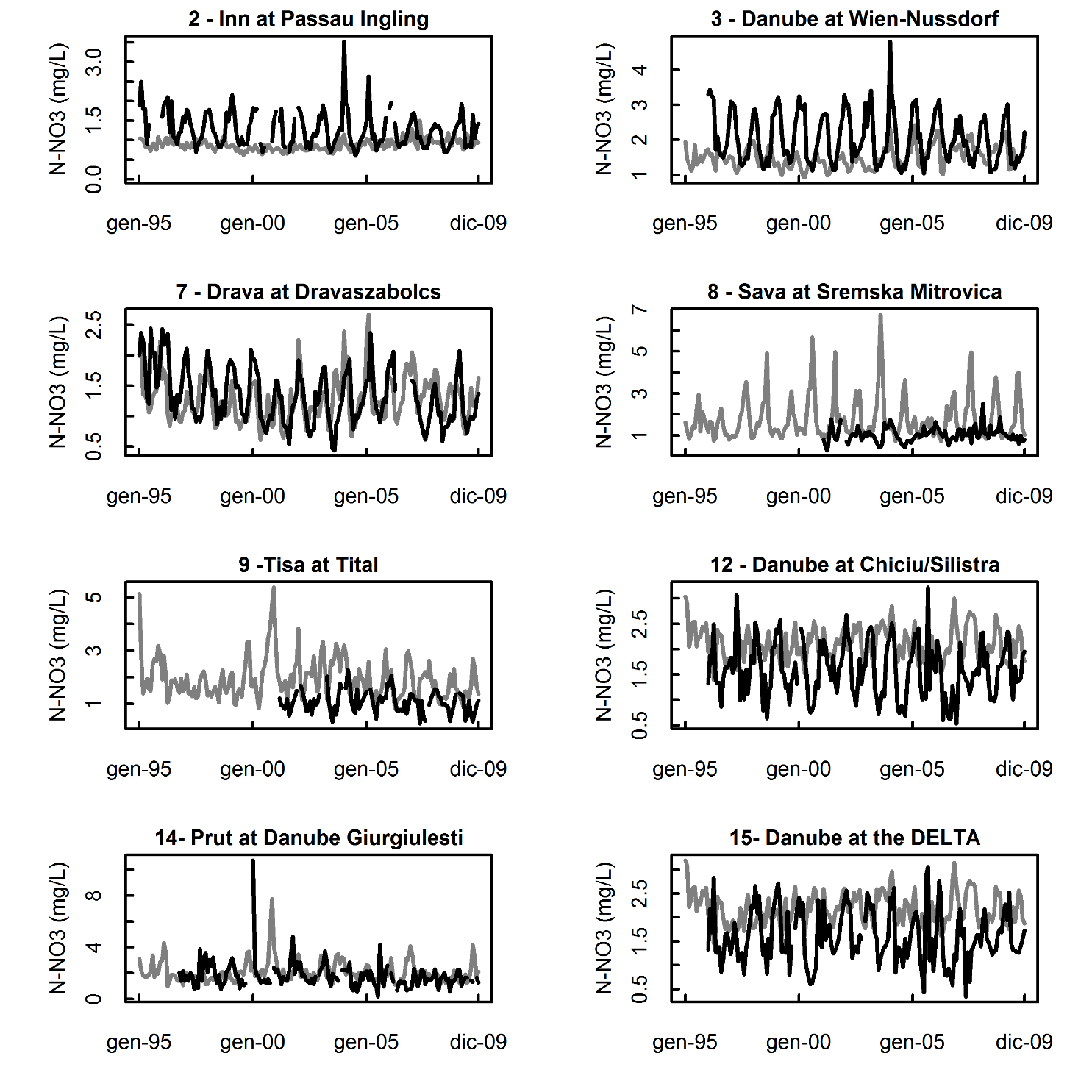


Figure S. 9. Monthly time series nitrogen nitrates N-NO3 (mg/L) as observed at the outlet of some water management regions (black line) and as simulated by SWAT model (grey line) for the period 1995-2009. Note the differences in y axis.


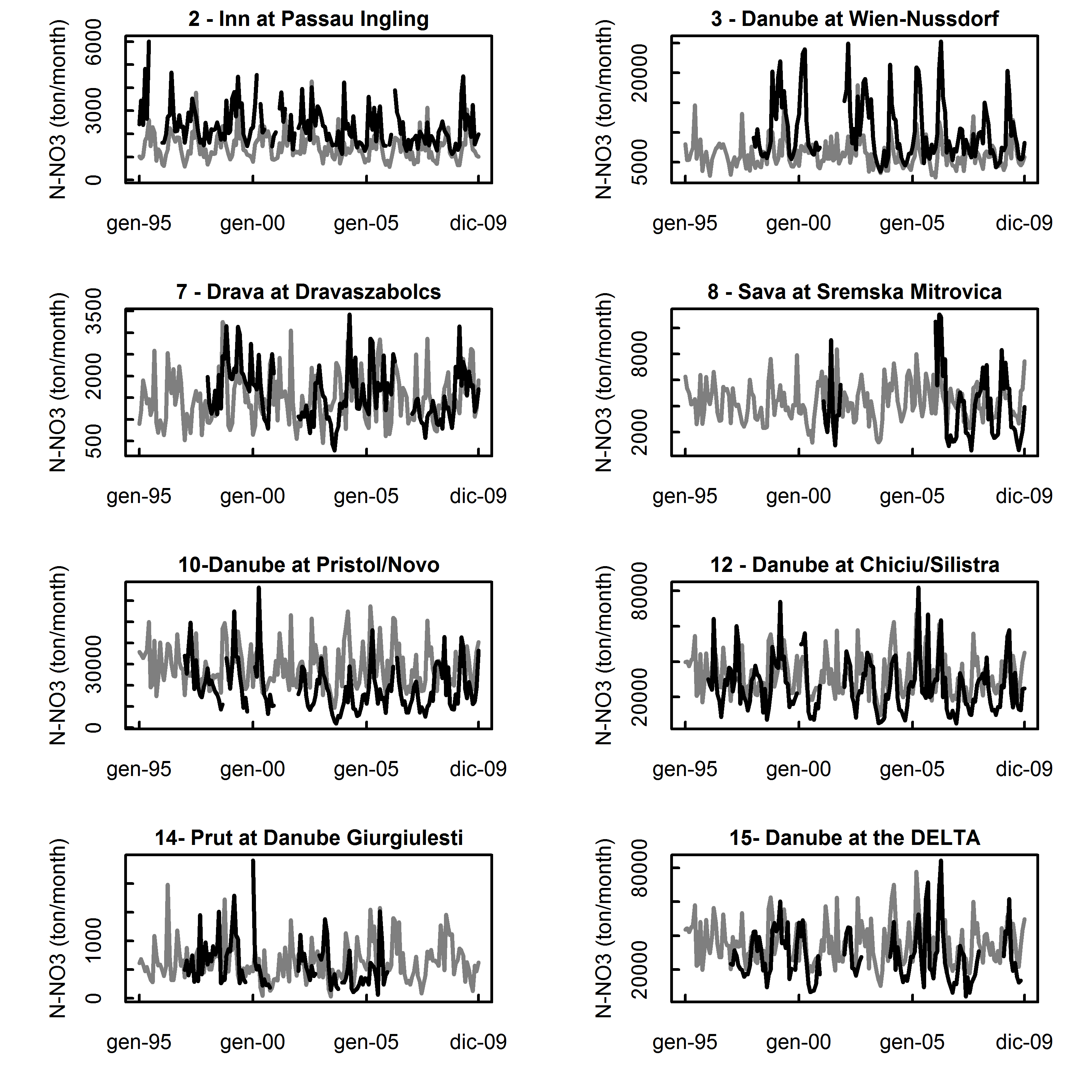


Figure S. 10. Monthly time series of nitrogen nitrates N-NO3 loads (ton) as observed at the outlet of some water management regions (black line) and as simulated by SWAT model (grey line) for the period 1995-2009. Note the differences in y axis.


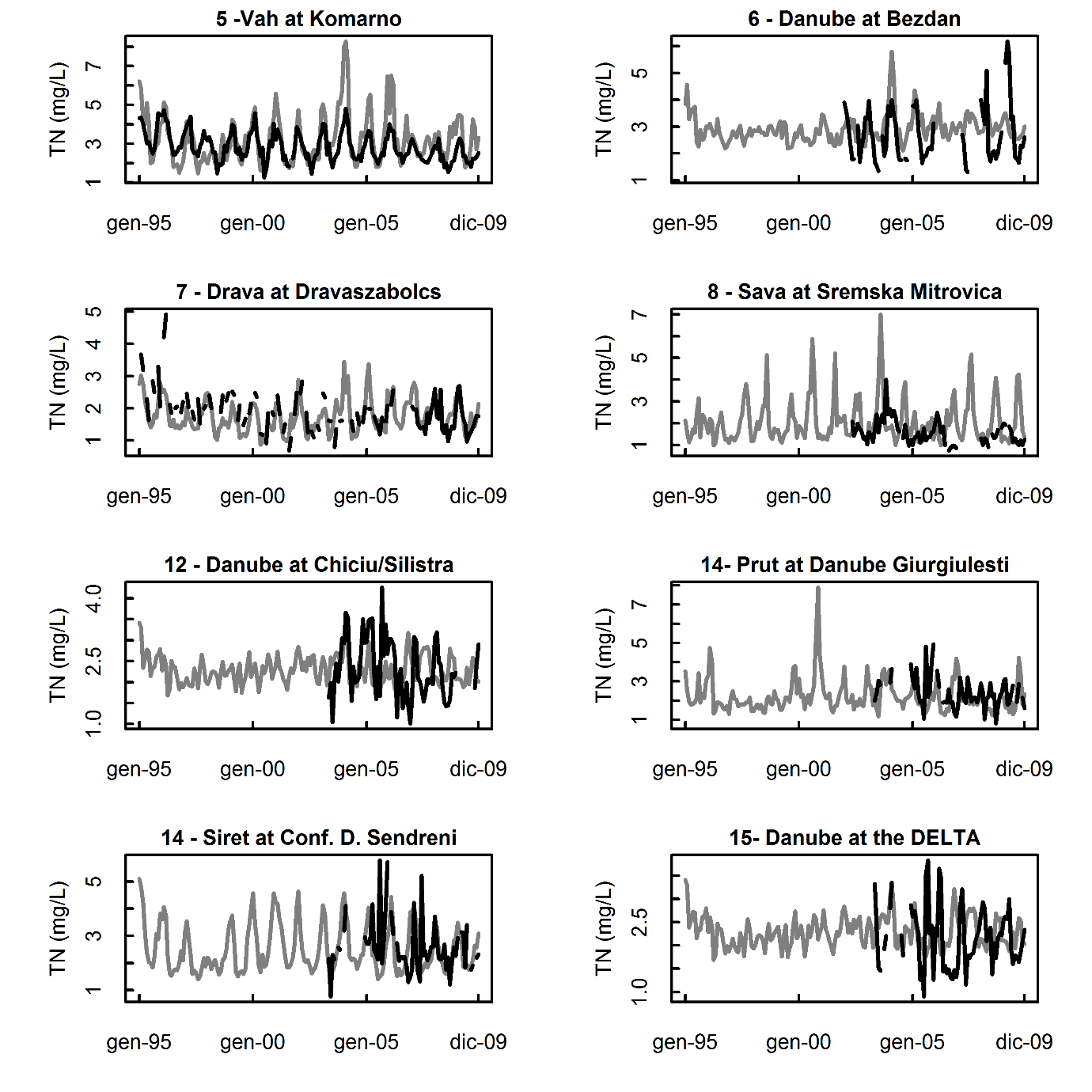


Figure S. 11. Monthly time series nitrogen nitrates TN (mg/L) as observed at the outlet of some water management regions (black line) and as simulated by SWAT model (grey line) for the period 1995-2009. Note the differences in y axis.


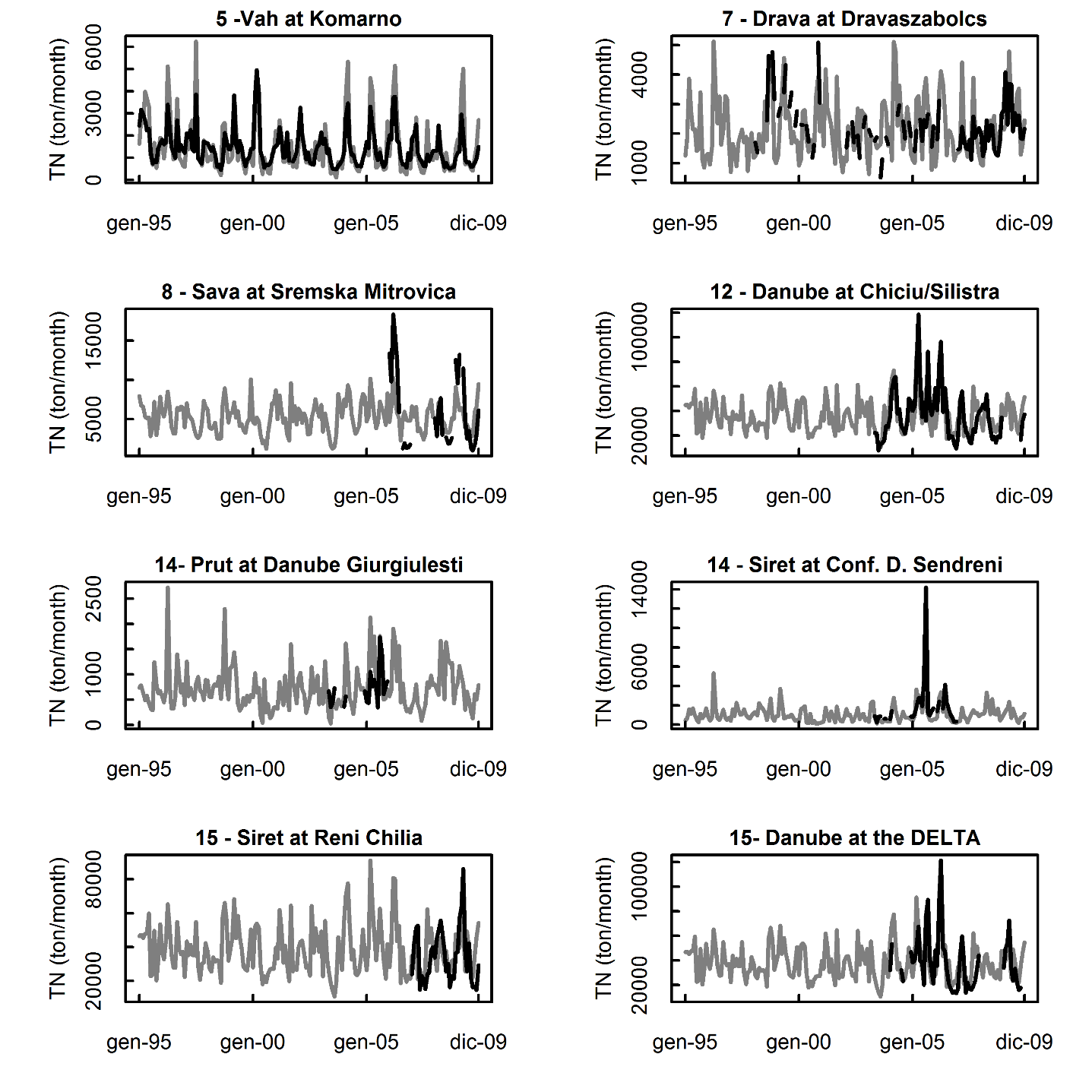


Figure S. 12. Monthly time series of total nitrogen TN loads (ton) as observed at the outlet of some water management regions (black line) and as simulated by SWAT model (grey line) for the period 1995-2009. Note the differences in y axis.


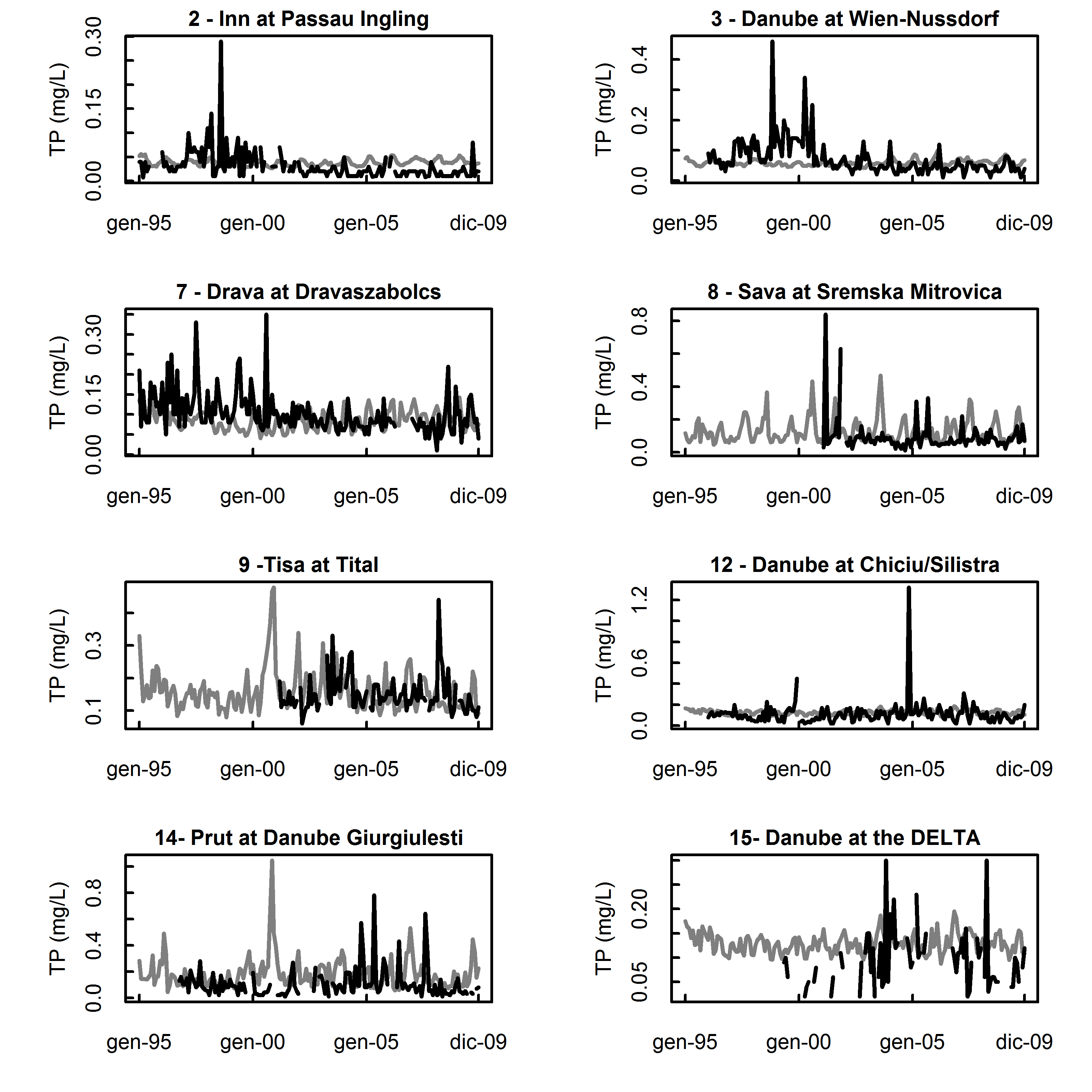


Figure S. 13. Monthly time series nitrogen nitrates TP (mg/L) as observed at the outlet of some water management regions (black line) and as simulated by SWAT model (grey line) for the period 1995-2009. Note the differences in y axis.


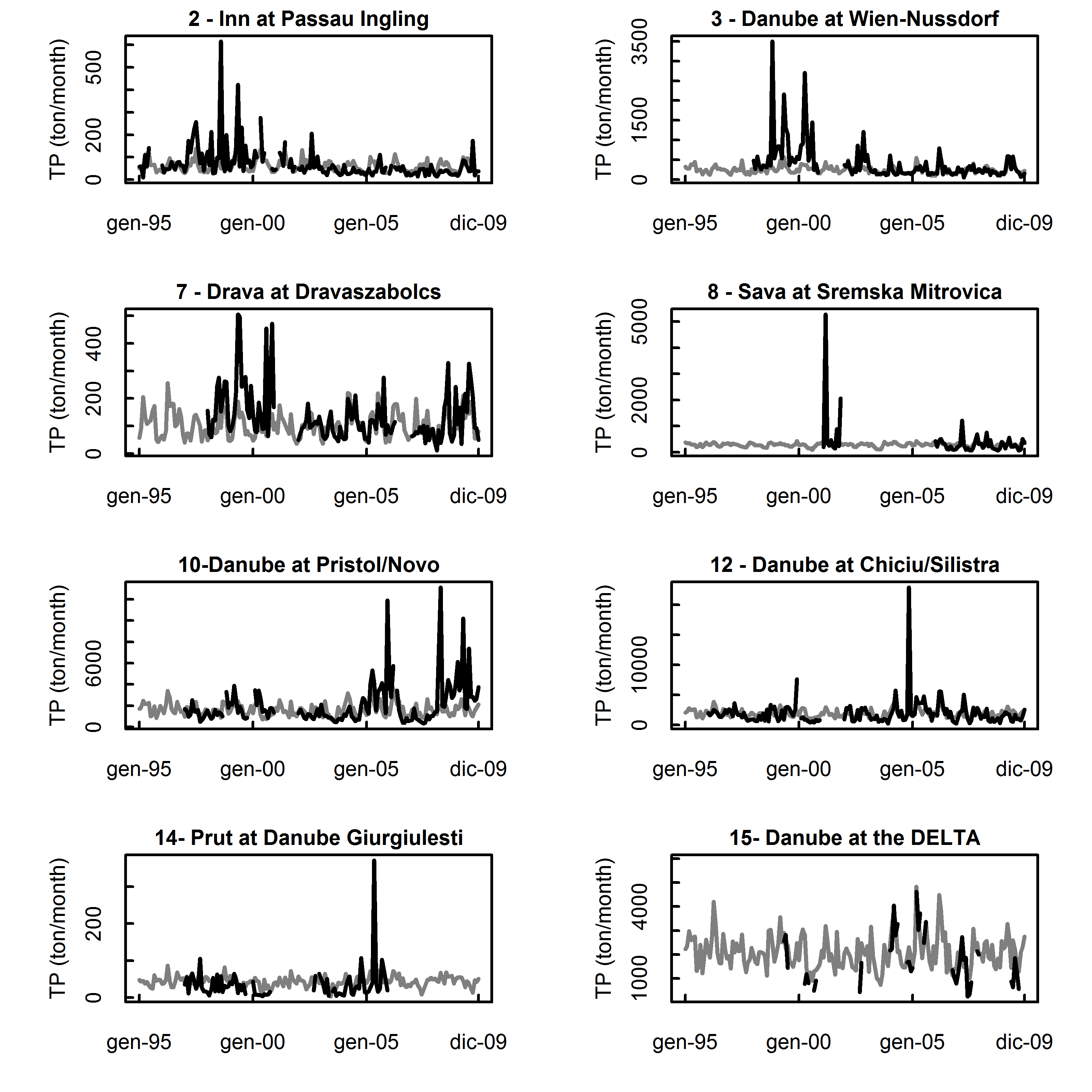


Figure S. 14. Monthly time series of total phosphorus TP loads (ton) as observed at the outlet of some water management regions (black line) and as simulated by SWAT model (grey line) for the period 1995-2009. Note the differences in y axis


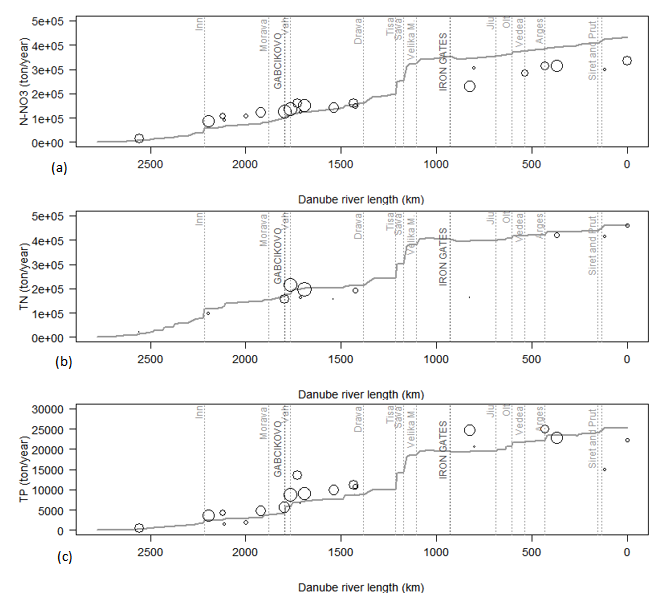


Figure S. 15. Long term average of annual nutrient loads (a,b,c) along the Danube River with SWAT for the period 1995-2009 together with the available observations recorded at the gauging stations. The observations are represented with bubbles with size proportional to the number of total observations in the simulated period (1995-2009). The confluences of main tributaries, as well as the main barriers are indicated.


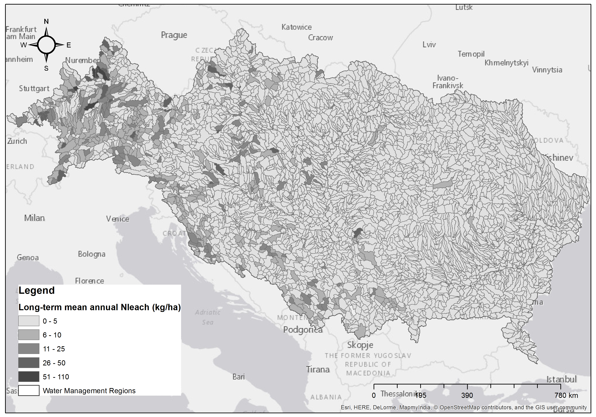


Figure S. 16. Spatial distribution of long-term mean annual nitrogen that leached into the aquifer

In the Danube River Basin.


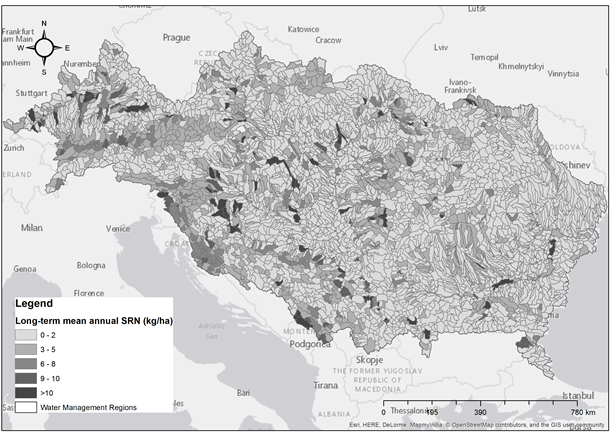


Figure S. 17. Spatial distribution of long-term mean annual nitrogen-nitrates loading to the reach in surface in the Danube River Basin.


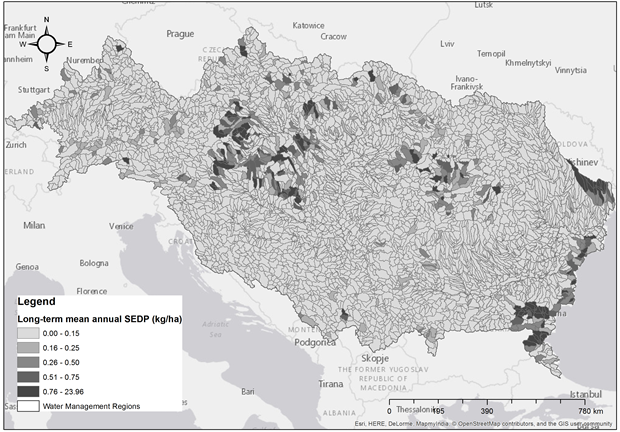


Figure S. 18. Spatial distribution of long-term mean annual mineral phosphorus adsorbed to sediment and transported into the reach in the Danube River Basin


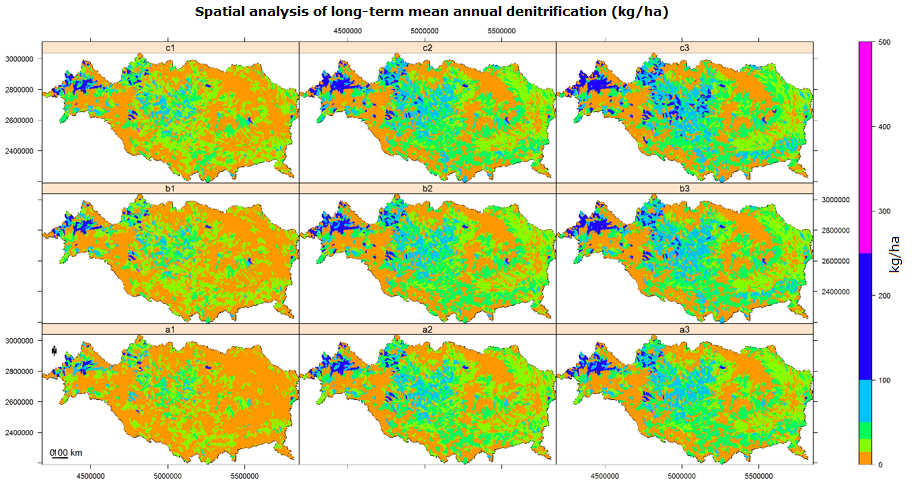


Figure S. 19. Spatial variation of long-term mean annual denitrification (kg/ha) in the Danube River Basin (period 1995-2009) with 9 combinations of CDN and SDNCO. Combinations: a1: CDN=0.6; SDNCO=1, a2: CDN=0.6; SDNCO=0.9, a3: CDN=0.6; SDNCO=0.8, b1: CDN=1.4; SDNCO=1, b2: CDN=1.4; SDNCO=0.9, b3: CDN=1.4; SDNCO=0.8, c1: CDN=3; SDNCO=1, c2: CDN=3; SDNCO=0.9, c3: CDN=3; SDNCO=0.8.


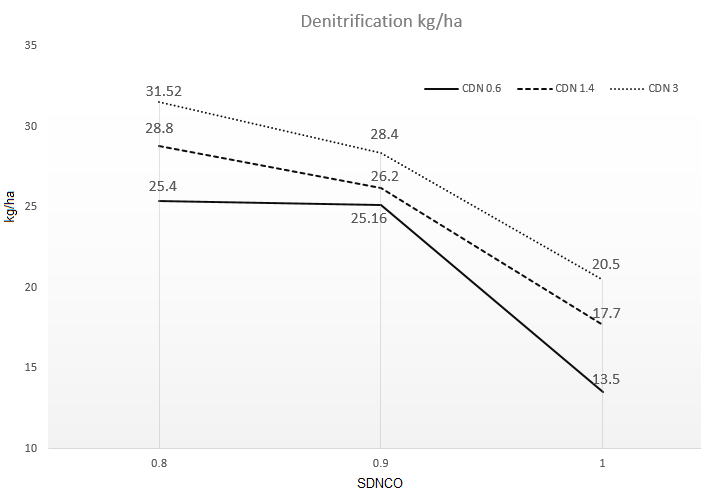


Figure S. 20. Long-term mean annual denitrification values (kg/ha, period 1995-2009) in the Danube River Basin with 9 combinations of CDN and SDNCO.

Table S1. Amount of fertilizers, nitrogen fixation, nitrogen in rain and nutrient discharges from point sources (PS) in each ICPDR water management region of the Danube Basin: N_APP_: total nitrogen (TN) fertilizer applied; NO3_APP_: Nitrate fertilizer applied; NH3_APP_: ammonia fertilizer applied; ORGN_APP_: organic nitrogen applied; P_APP_: total phosphorus (TP) fertilizer applied; SOLP_APP_: mineral phosphorus applied; ORGP_APP_: organic phosphorus applied; N_RAIN_: nitrate added to soil profile by rain; N_FIX_: nitrogen fixation.

| ID | Name | N_APP_ | NO3_APP_ | NH3_APP_ | ORGN_APP_ | P_APP_ | SOLP_APP_ | ORGP_APP_ | N_RAIN_ | N_FIX_ | TN from PS | TP from PS |
| --- | --- | --- | --- | --- | --- | --- | --- | --- | --- | --- | --- | --- |
|  |  | kg/ha | % | % | % | kg/ha | % | % | kg/ha | kg/ha | kton/y | kton/y |
| 1 | Upper Danube | 105 | 54 | 8 | 38 | 14 | 73 | 27 | 17 | 19 | 15 | 1 |
| 2 | Inn | 21 | 48 | 10 | 43 | 4 | 77 | 24 | 22 | 35 | 6 | 0 |
| 3 | Austrian Danube | 27 | 54 | 8 | 38 | 6 | 85 | 16 | 18 | 37 | 6 | 0 |
| 4 | Morava | 78 | 66 | 6 | 28 | 10 | 82 | 20 | 11 | 18 | 5 | 0 |
| 5 | Vah-Hron-Ipel | 33 | 61 | 7 | 32 | 5 | 83 | 19 | 13 | 27 | 7 | 1 |
| 6 | Pannonian Danube | 69 | 68 | 6 | 27 | 10 | 84 | 18 | 11 | 21 | 23 | 2 |
| 7 | Drava | 29 | 59 | 8 | 34 | 5 | 83 | 18 | 16 | 47 | 10 | 1 |
| 8 | Sava | 31 | 50 | 9 | 42 | 5 | 80 | 24 | 18 | 37 | 43 | 4 |
| 9 | Tisa | 37 | 61 | 7 | 33 | 5 | 81 | 22 | 12 | 25 | 26 | 3 |
| 10 | Middle Danube | 34 | 57 | 8 | 36 | 6 | 85 | 21 | 12 | 40 | 10 | 1 |
| 11 | Velika Morava | 34 | 56 | 8 | 36 | 5 | 78 | 26 | 13 | 38 | 10 | 1 |
| 12 | Bulgarian Danube | 31 | 65 | 7 | 29 | 4 | 82 | 22 | 11 | 23 | 10 | 1 |
| 13 | Romanian Danube | 19 | 58 | 8 | 35 | 4 | 87 | 17 | 11 | 34 | 28 | 4 |
| 14 | Siret-Prut | 16 | 46 | 10 | 46 | 3 | 82 | 21 | 11 | 36 | 12 | 2 |
| 15 | Delta-Liman | 19 | 31 | 13 | 58 | 4 | 80 | 26 | 8 | 22 | 1 | 0 |
|  | Danube15^d^ | 39 | 55 | 8 | 37 | 6 | 81 | 21 | 13 | 31 | 214 | 21 |

d. Danube15 refers to the whole drained area of the 15 regions
